# Supplementary material for: The implications of outcome truncation in reproductive medicine RCTs: a simulation platform for trialists and simulation study
Source: Trials. 2021 Aug 6;22:520. doi: 10.1186/s13063-021-05482-4 (PMC8344218; doi:10.1186/s13063-021-05482-4)
Supplement: Supplementary file 1 — Additional file 1: Supplementary Figures. Figures corresponding to additional results from the simulation study, including sensitivity analyses. [file 13063_2021_5482_MOESM1_ESM.docx]

Supplementary Figures

Contents

[Continuous outcome study 2](#_Toc74672231)

[Bias 2](#_Toc74672232)

[Coverage 5](#_Toc74672233)

[Type 1 error 8](#_Toc74672234)

[Empirical SE 11](#_Toc74672235)

[Model SE 15](#_Toc74672236)

[Binary outcomes 19](#_Toc74672237)

[Missing data 19](#_Toc74672238)

[Bias 23](#_Toc74672239)

[Coverage 25](#_Toc74672240)

[Empirical SE 27](#_Toc74672241)

[Model SE 29](#_Toc74672242)

[Type 1 error 31](#_Toc74672243)

# Continuous outcome study

## Bias


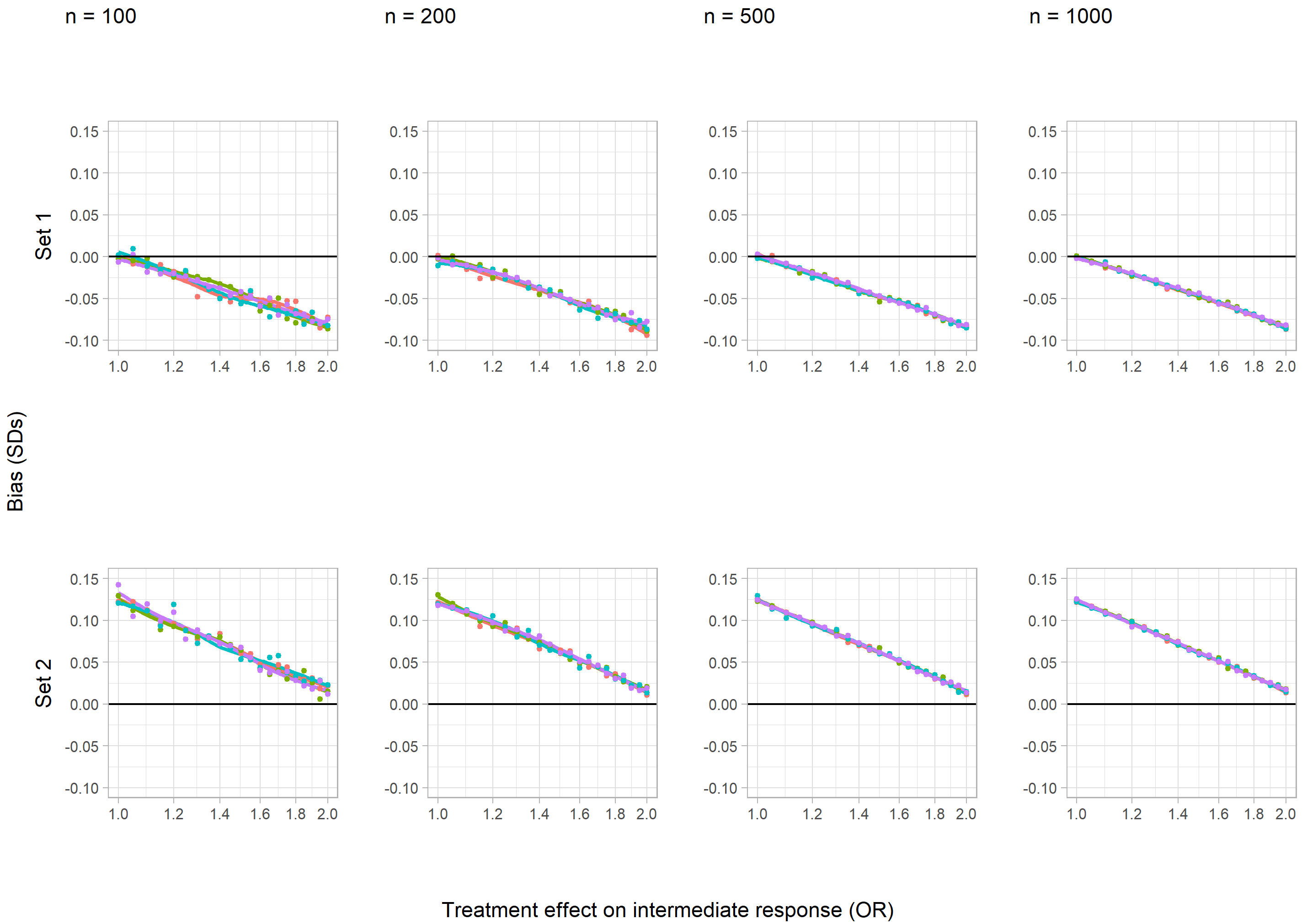


S Figure 1: Bias of a simple difference in means in the continuous outcome simulation study (sensitivity analysis A, increased confounding) in standard deviations of the outcome. Colour indicates treatment effect on the outcome variable (SDs): red = 0, green = 0.2, blue = 1, purple = 5.


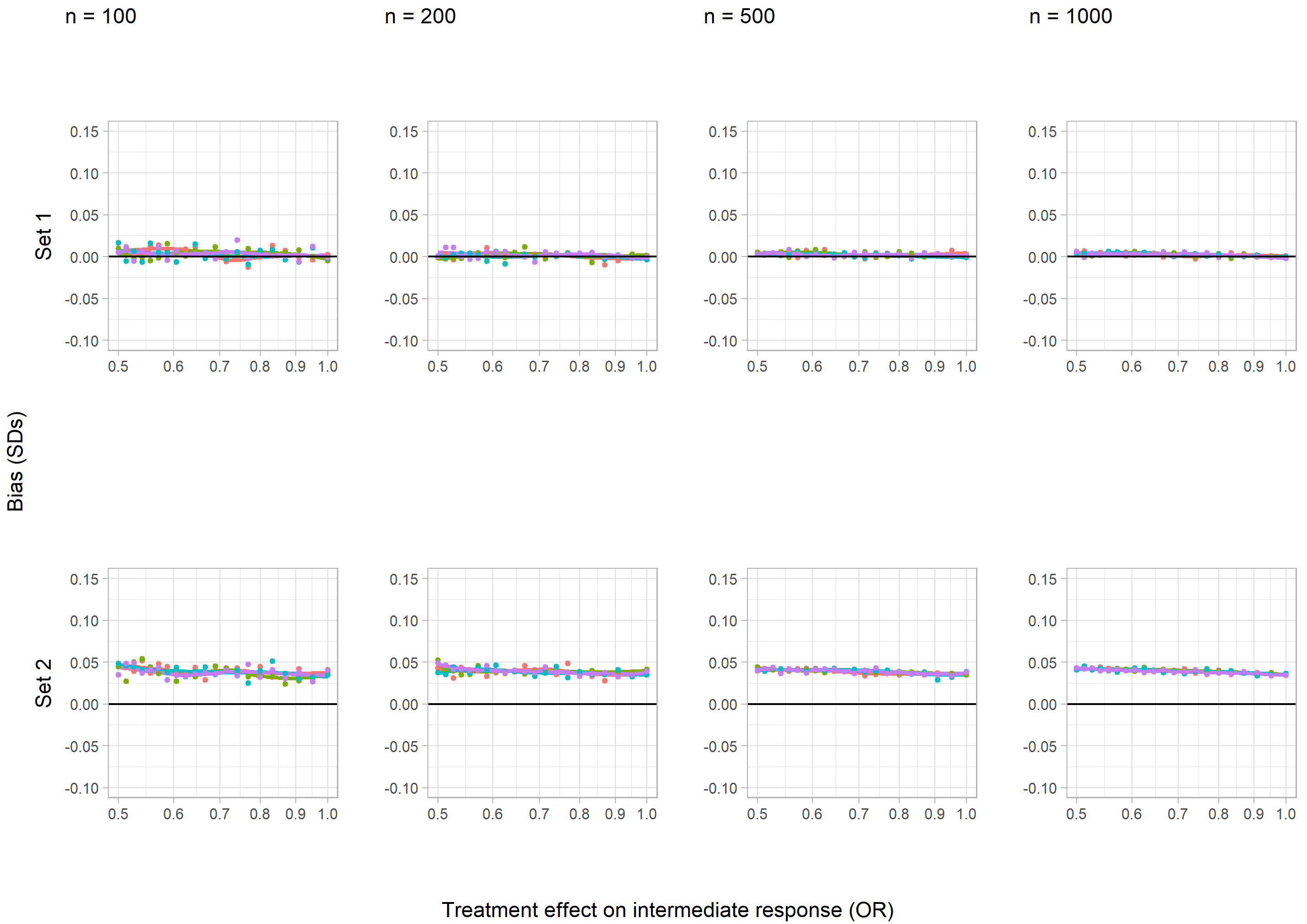


S Figure 2: Bias of a simple difference in means in the continuous outcome simulation study (sensitivity analysis B, changed direction of treatment effect on intermediate) in standard deviations of the outcome . Colour indicates treatment effect on the outcome variable (SDs): red = 0, green = 0.2, blue = 1, purple = 5.


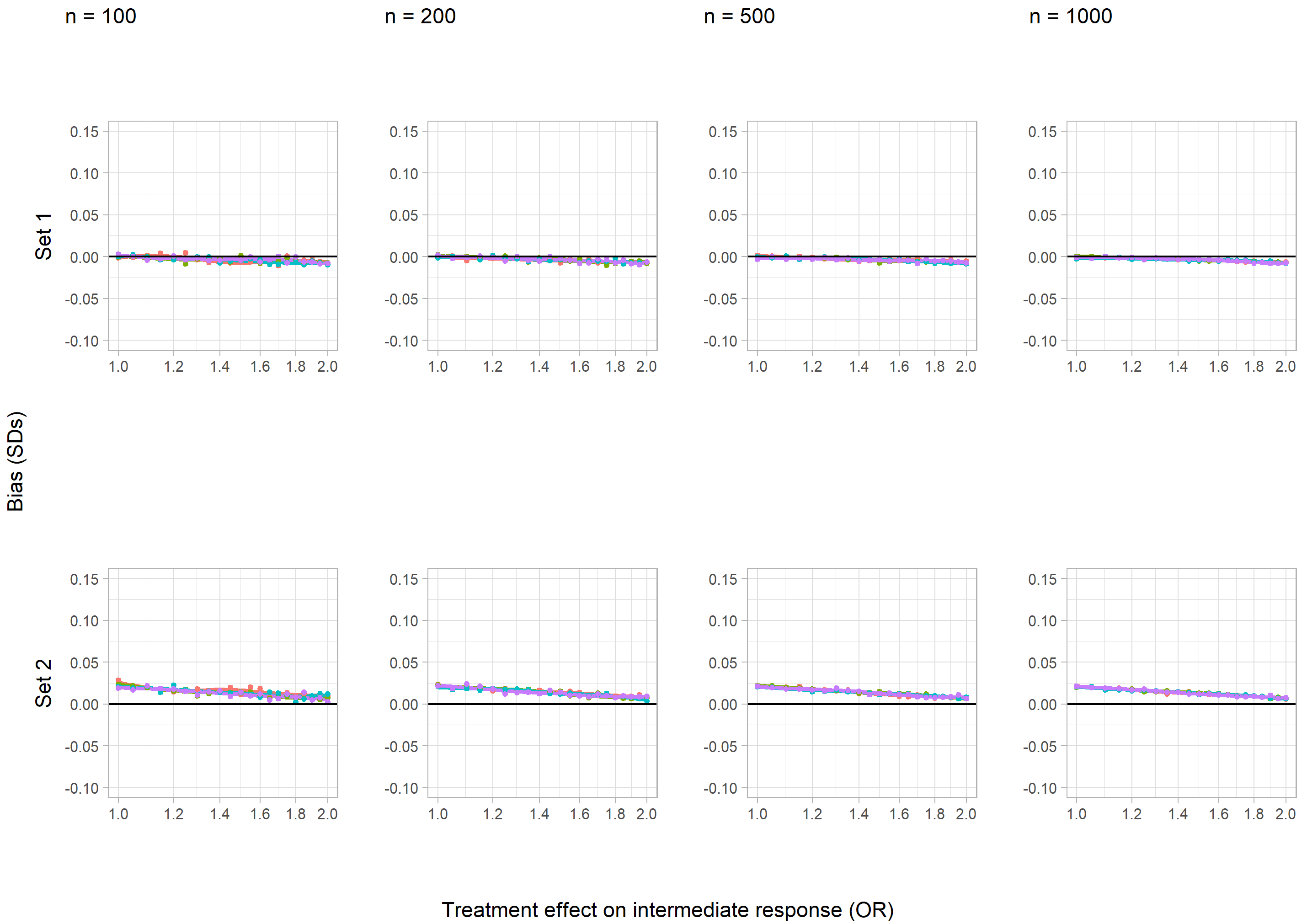


S Figure 3: Bias of a simple difference in means in the continuous outcome simulation study (sensitivity analysis C, increased event rate) in standard deviations of the outcome . Colour indicates treatment effect on the outcome variable (SDs): red = 0, green = 0.2, blue = 1, purple = 5.

## Coverage


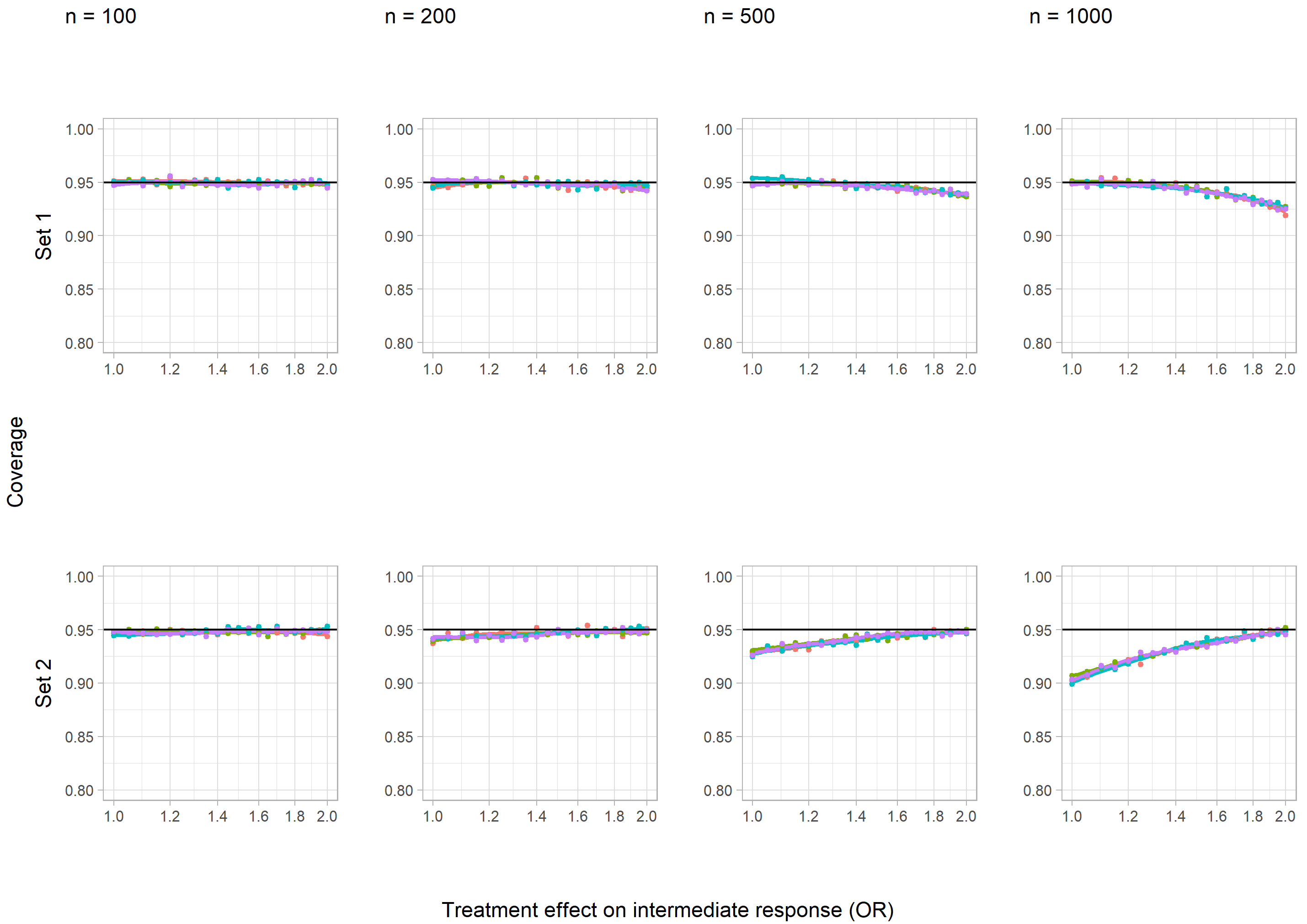


S Figure 4: Coverage of a 95% confidence interval corresponding to a simple difference in means in the continuous outcome simulation study (sensitivity analysis A), increased confounding). Colour indicates treatment effect on the outcome variable (SDs): red = 0, green = 0.2, blue = 1, purple = 5.


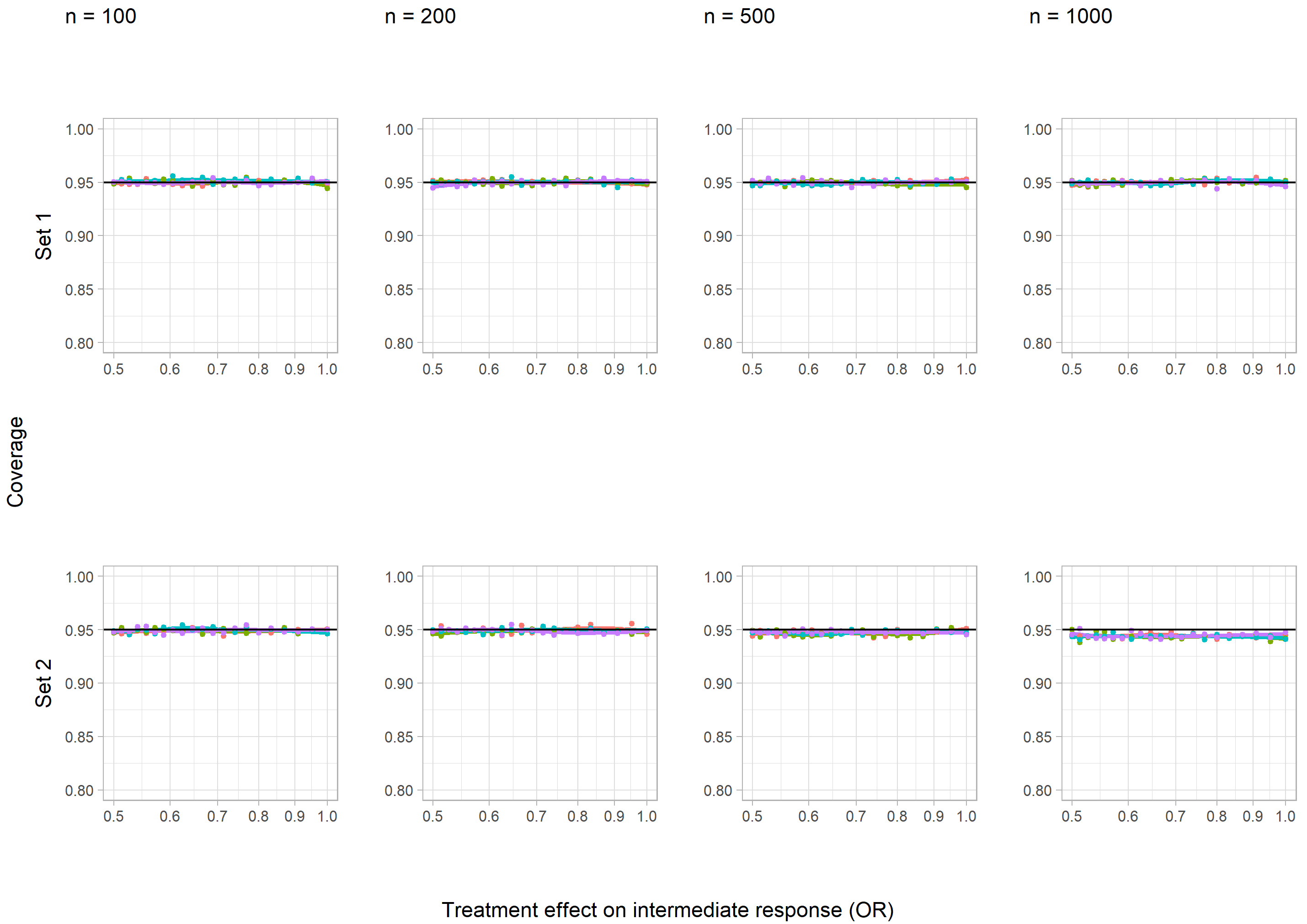


S Figure 5: Coverage of a 95% confidence interval corresponding to a simple difference in means in the continuous outcome simulation study (sensitivity analysis B) changed direction of treatment effect on intermediate). Colour indicates treatment effect on the outcome variable (SDs): red = 0, green = 0.2, blue = 1, purple = 5.


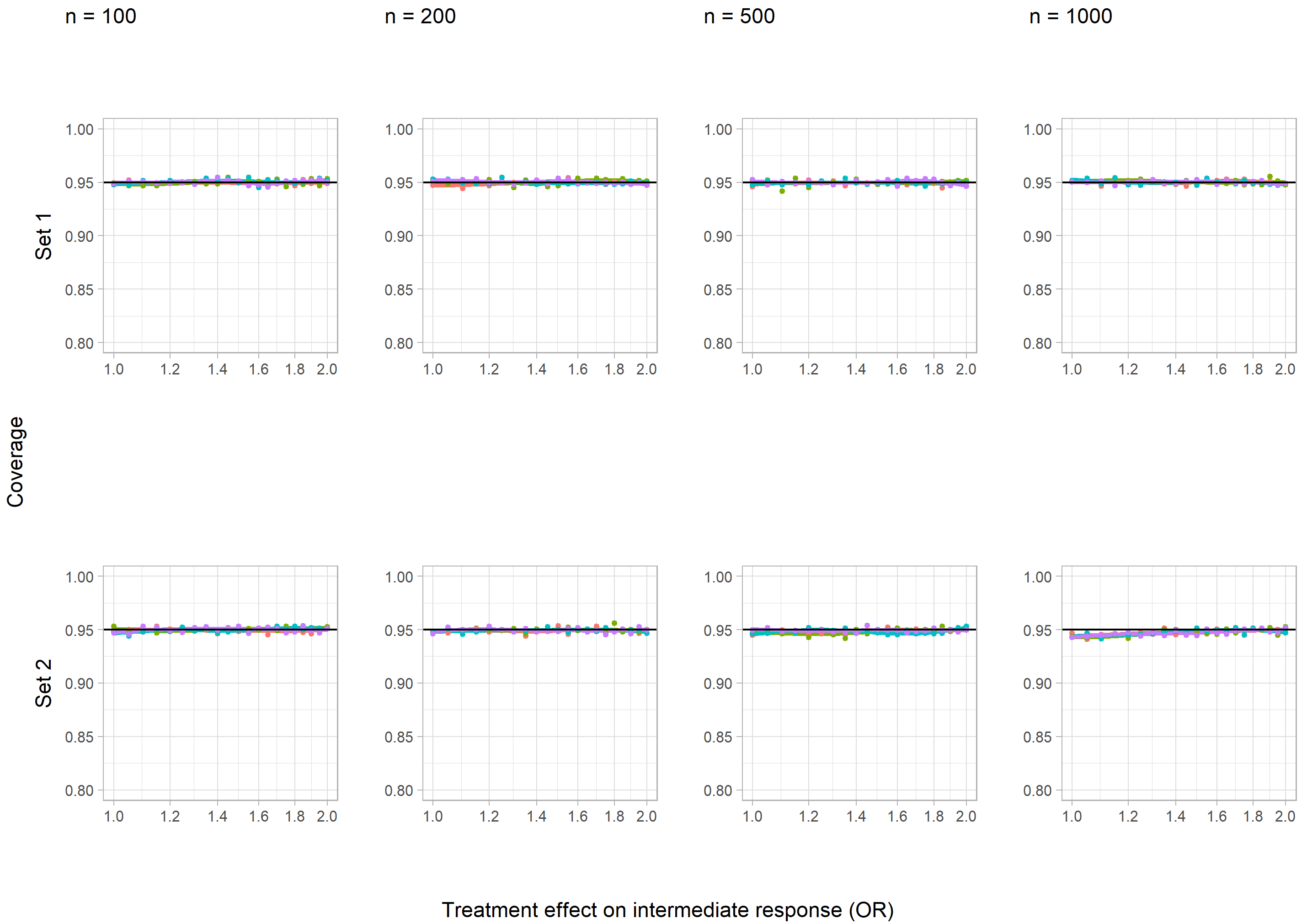


S Figure 6: Coverage of a 95% confidence interval corresponding to a simple difference in means in the continuous outcome simulation study (sensitivity analysis C), increase event rate). Colour indicates treatment effect on the outcome variable (SDs): red = 0, green = 0.2, blue = 1, purple = 5.

## Type 1 error


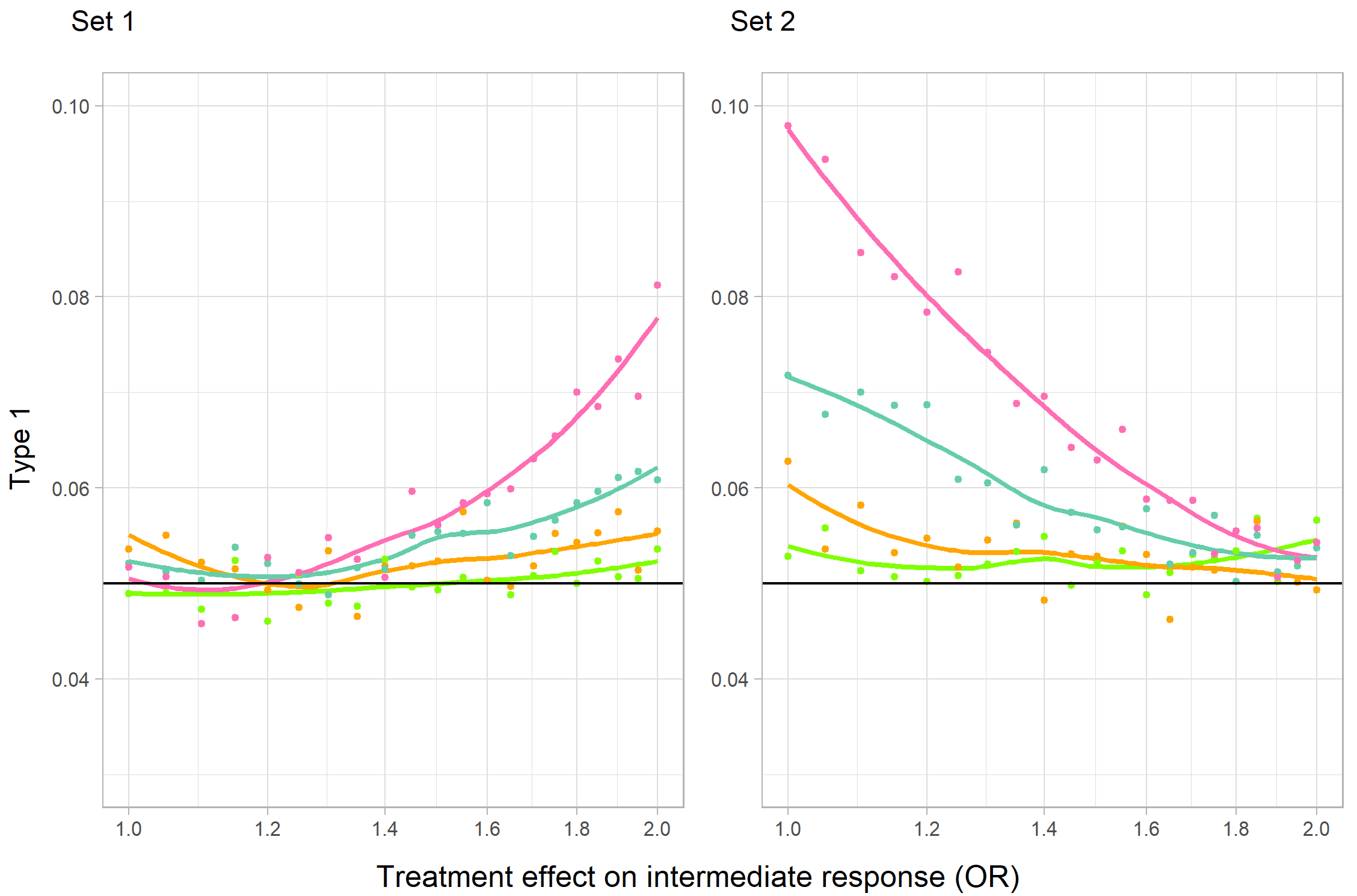


S Figure 7: Type 1 error of a t-test in the continuous outcome simulation study at a 5% significance level (sensitivity analysis A), increased confounding). Colour indicates the total starting sample size in each simulated trial (light green = 100, orange = 200, dark green = 500, pink = 1000). Horizontal line indicates the nominal level.


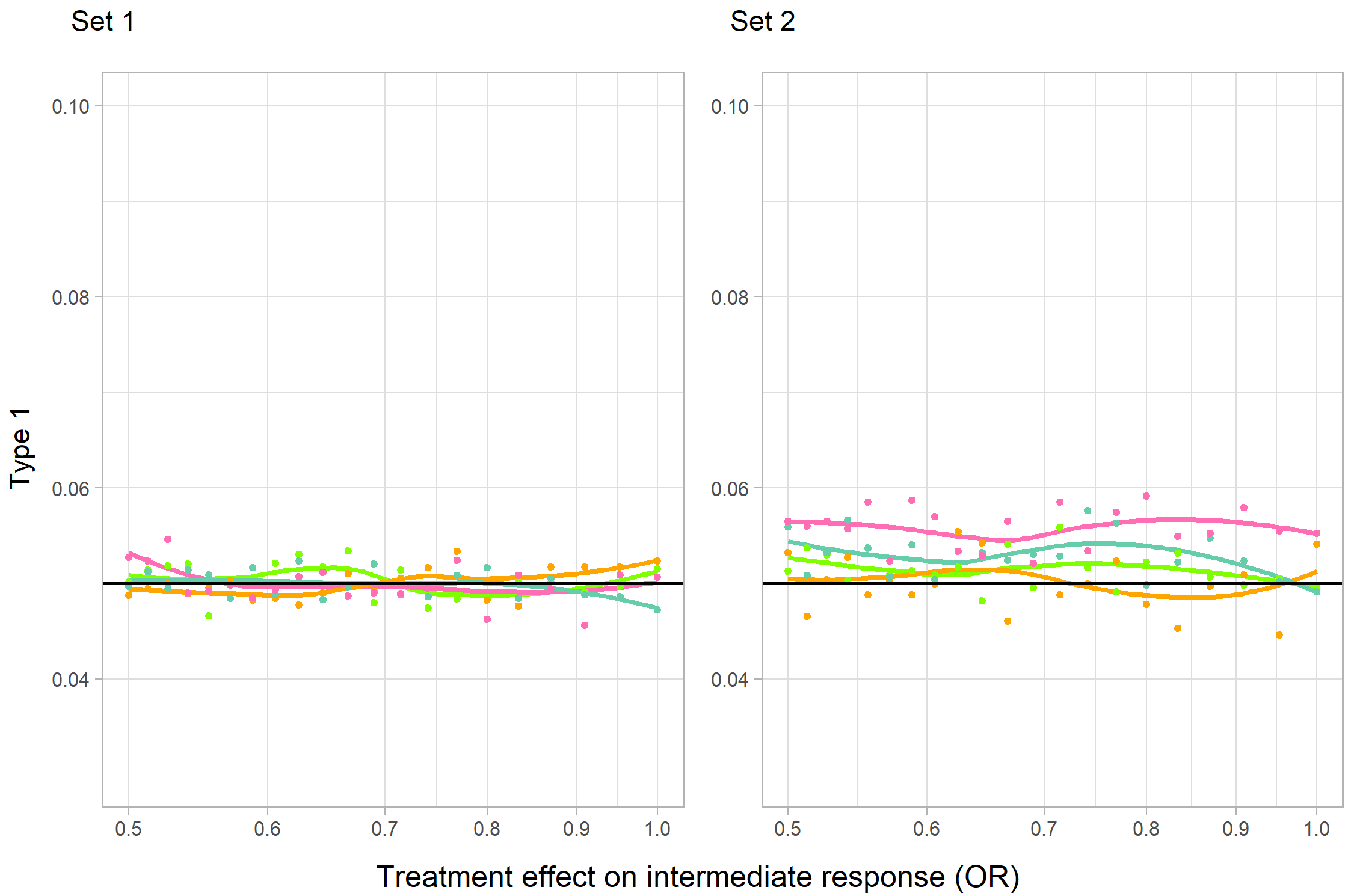


S Figure 8: Type 1 error of a t-test in the continuous outcome simulation study at a 5% significance level (sensitivity analysis B) changed direction of effect on intermediate). Colour indicates the total starting sample size in each simulated trial (light green = 100, orange = 200, dark green = 500, pink = 1000). Horizontal line indicates the nominal level.


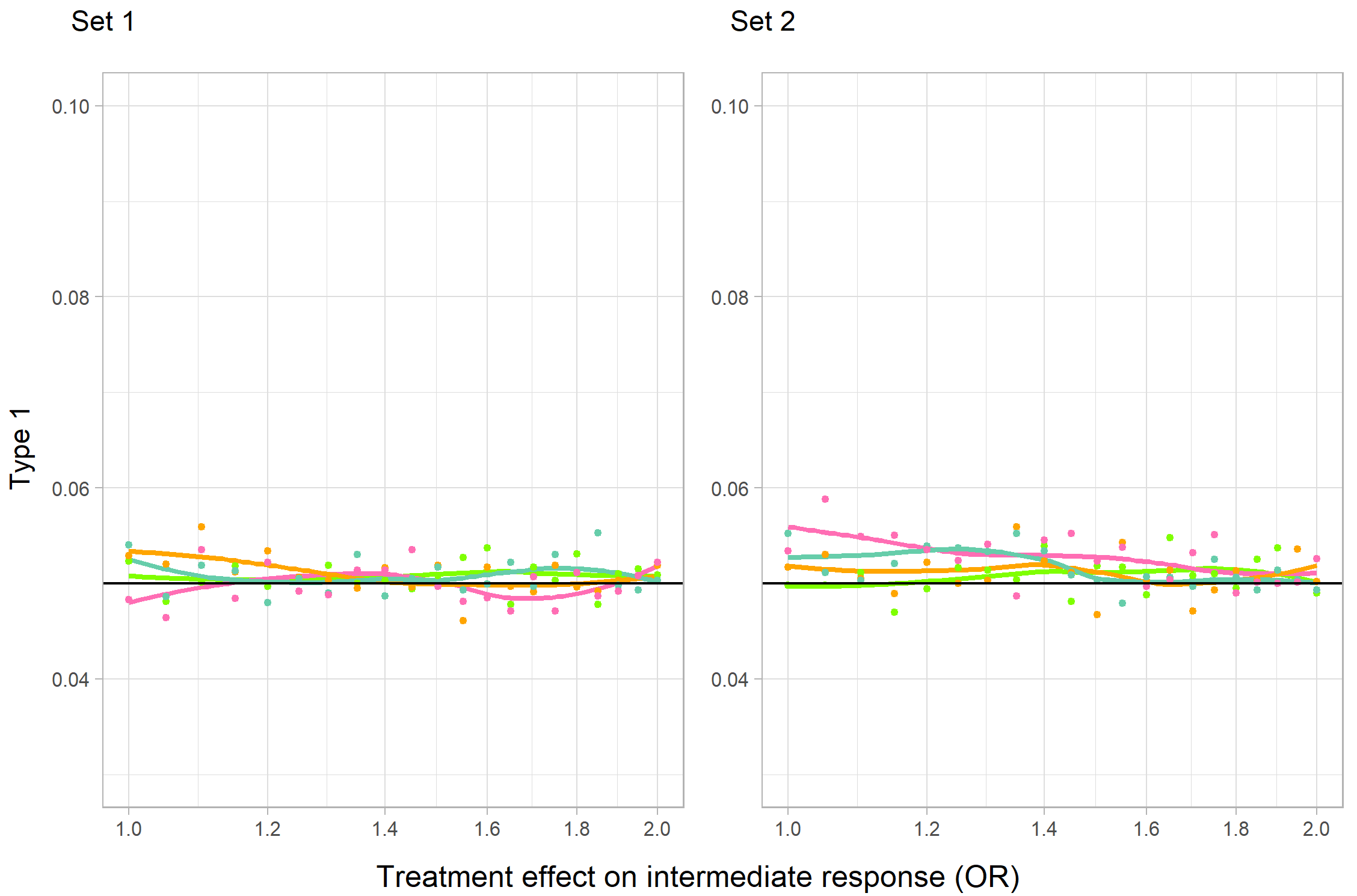


S Figure 9: Type 1 error of a t-test in the continuous outcome simulation study at a 5% significance level (sensitivity analysis C), increased event rate). Colour indicates the total starting sample size in each simulated trial (light green = 100, orange = 200, dark green = 500, pink = 1000). Horizontal line indicates the nominal level.

## Empirical SE


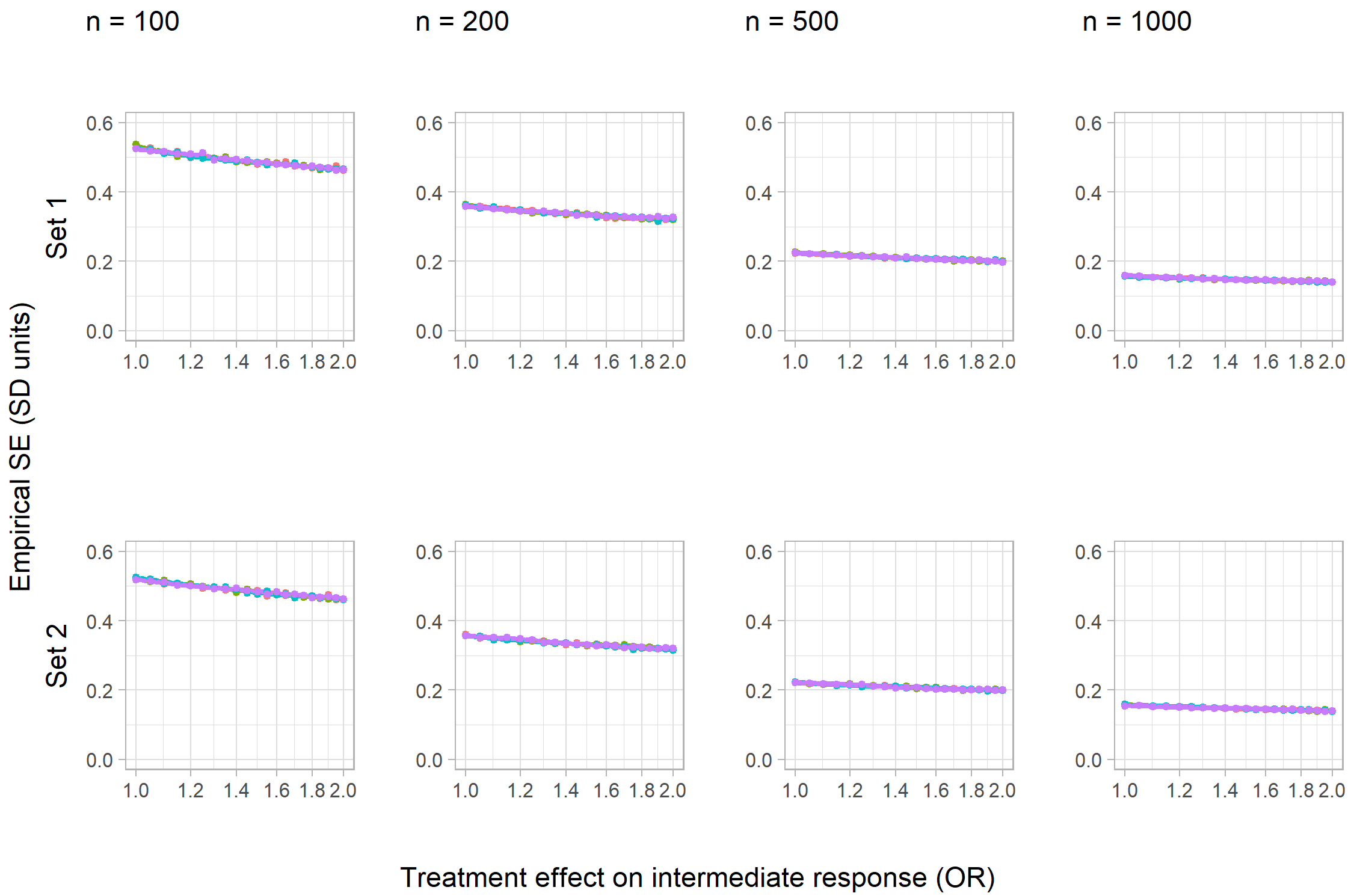


S Figure 10: Empirical SE of a simple difference in means in the continuous outcome study (core senarios). Colour indicates treatment effect on the outcome variable (SDs): red = 0, green = 0.2, blue = 1, purple = 5.


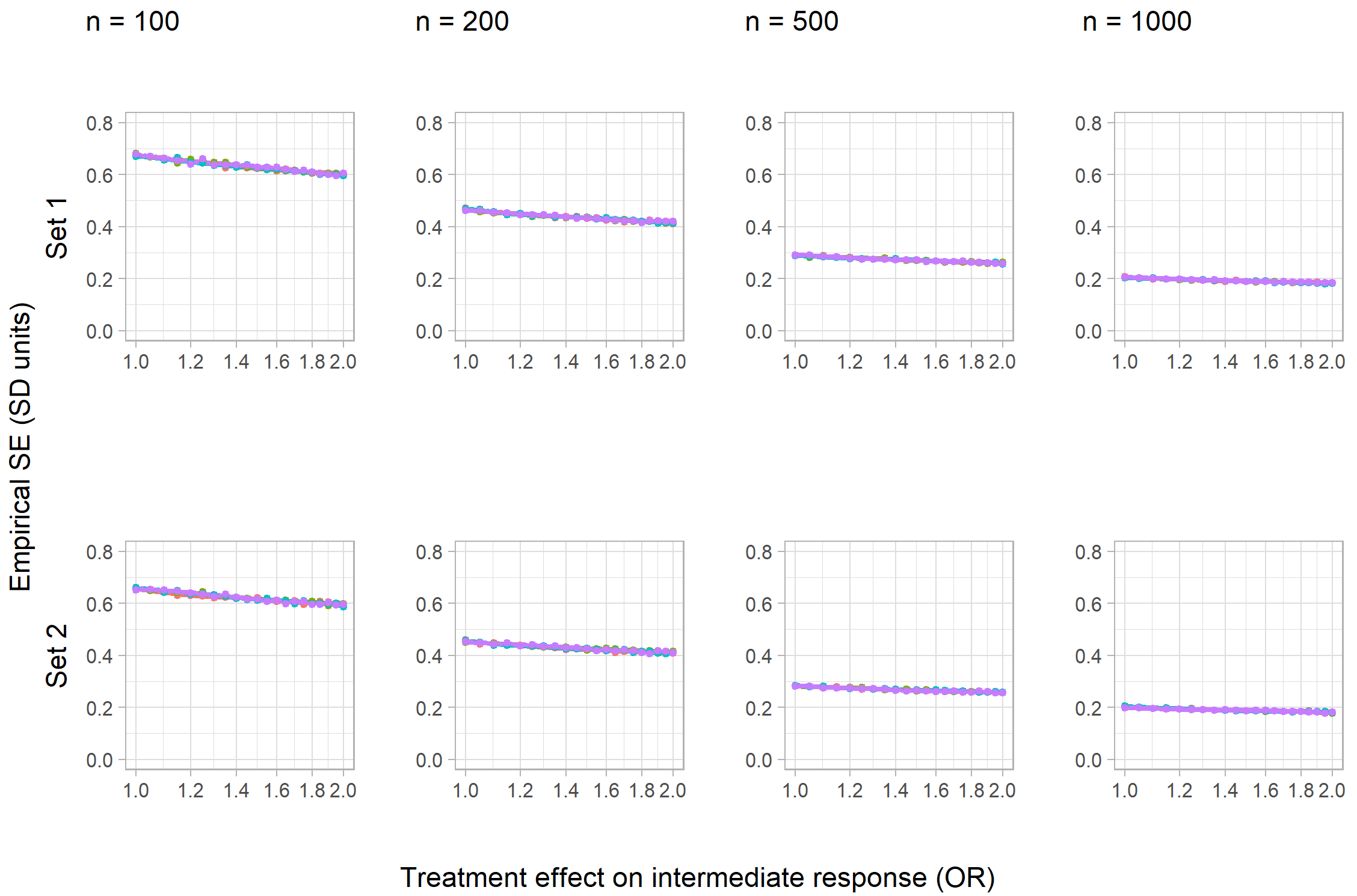


S Figure 11: Empirical SE of a simple difference in means in the continuous outcome study (sensitivity analysis A), increased confounding). Colour indicates treatment effect on the outcome variable (SDs): red = 0, green = 0.2, blue = 1, purple = 5.


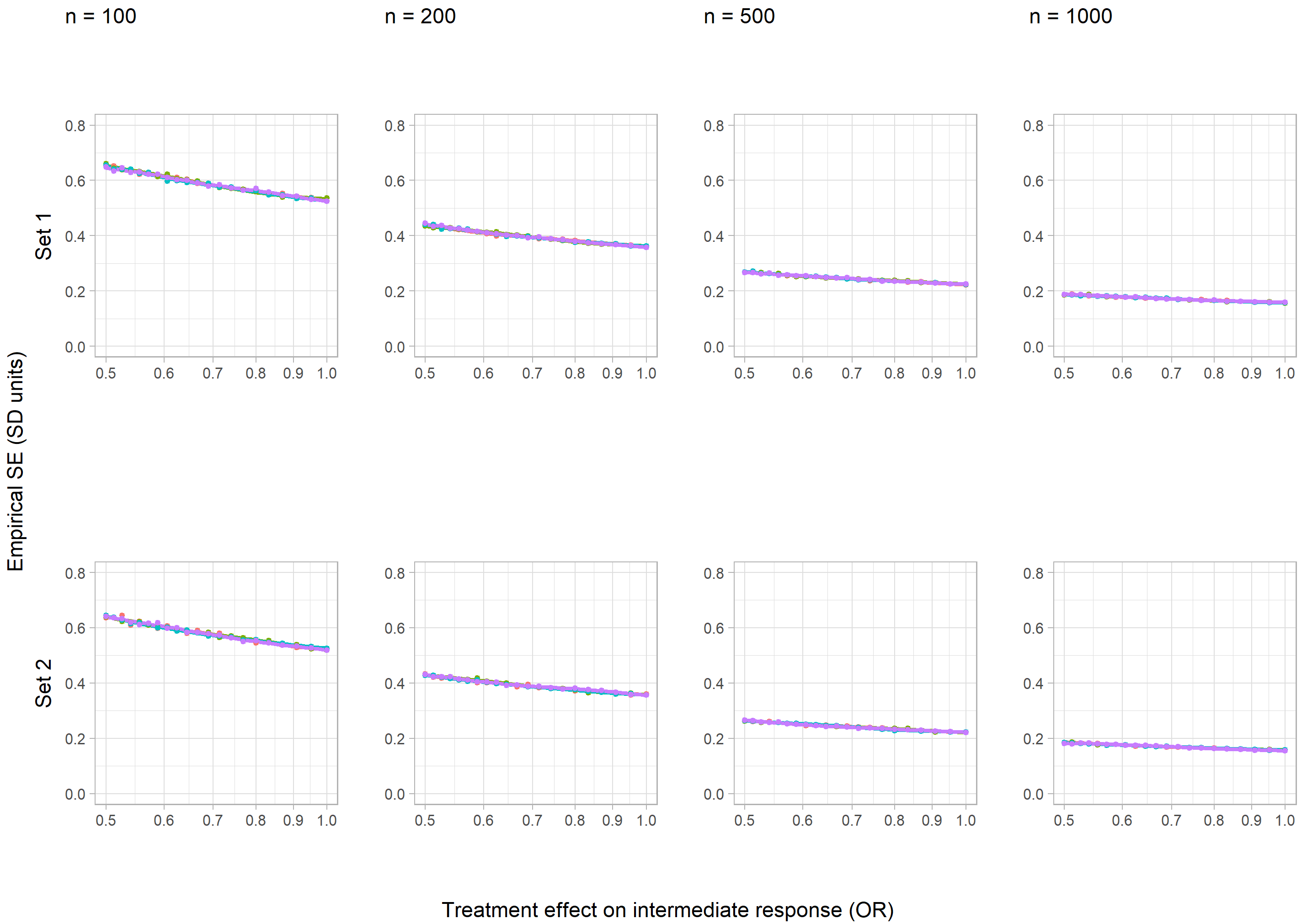


S Figure 12: Empirical SE of a simple difference in means in the continuous outcome study (sensitivity analysis B), changed direction of treatment effect on intermediate). Colour indicates treatment effect on the outcome variable (SDs): red = 0, green = 0.2, blue = 1, purple = 5.


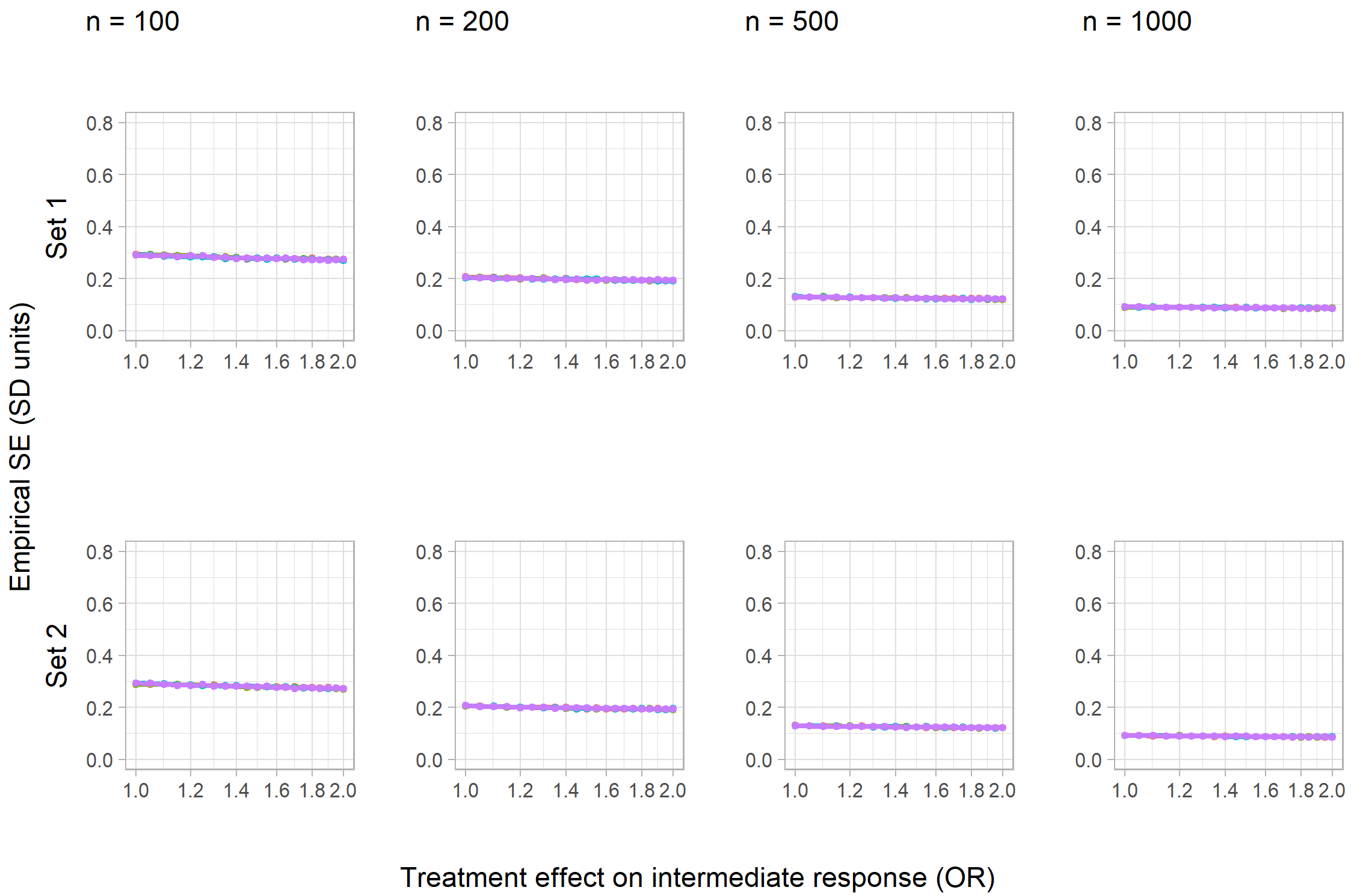


S Figure 13: Empirical SE of a simple difference in means in the continuous outcome study (sensitivity analysis C), increased event rate). Colour indicates treatment effect on the outcome variable (SDs): red = 0, green = 0.2, blue = 1, purple = 5.

## Model SE


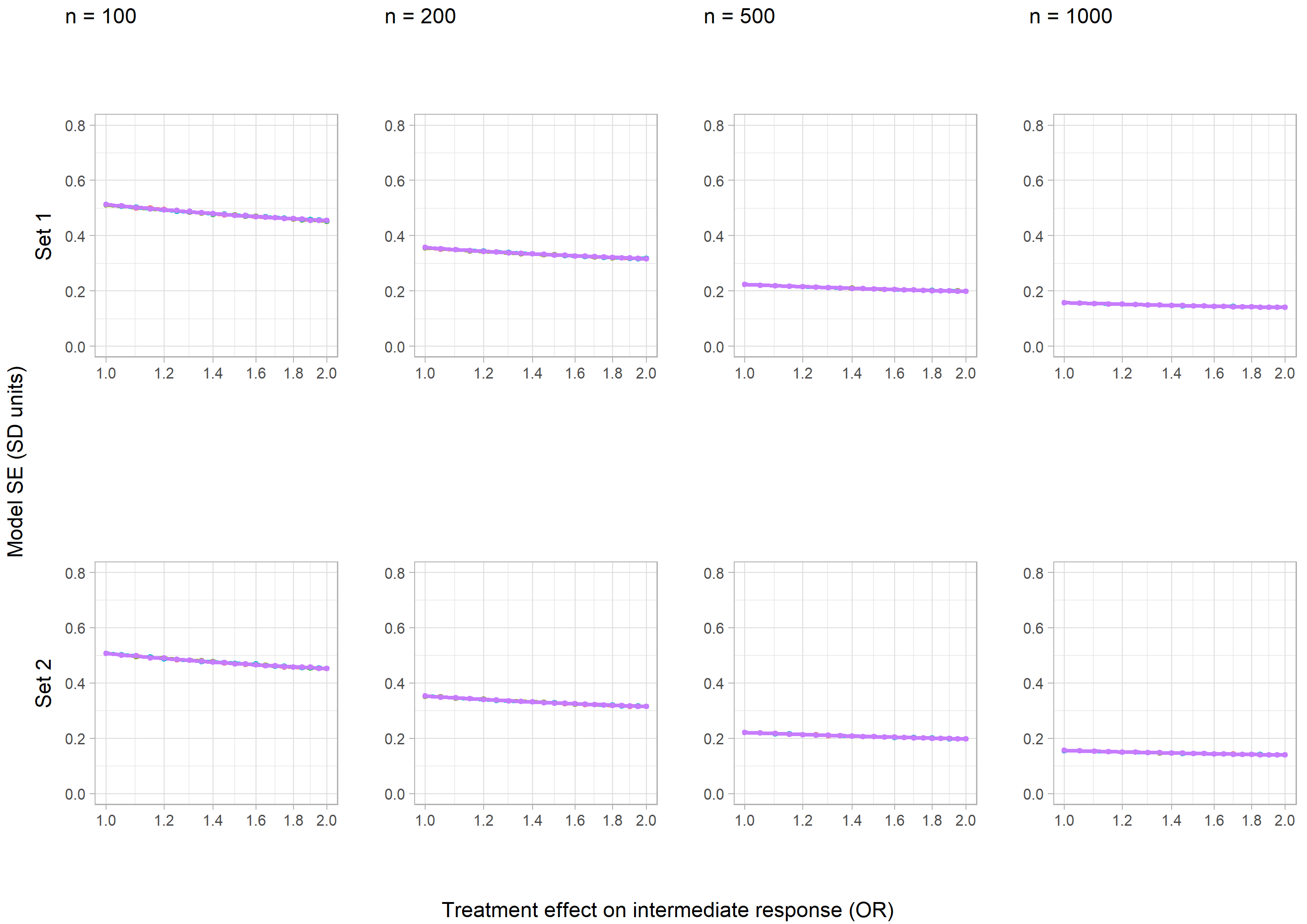


S Figure 14: Model SE of a simple difference in means in the continuous outcome study (core scenarios). Colour indicates treatment effect on the outcome variable (SDs): red = 0, green = 0.2, blue = 1, purple = 5.


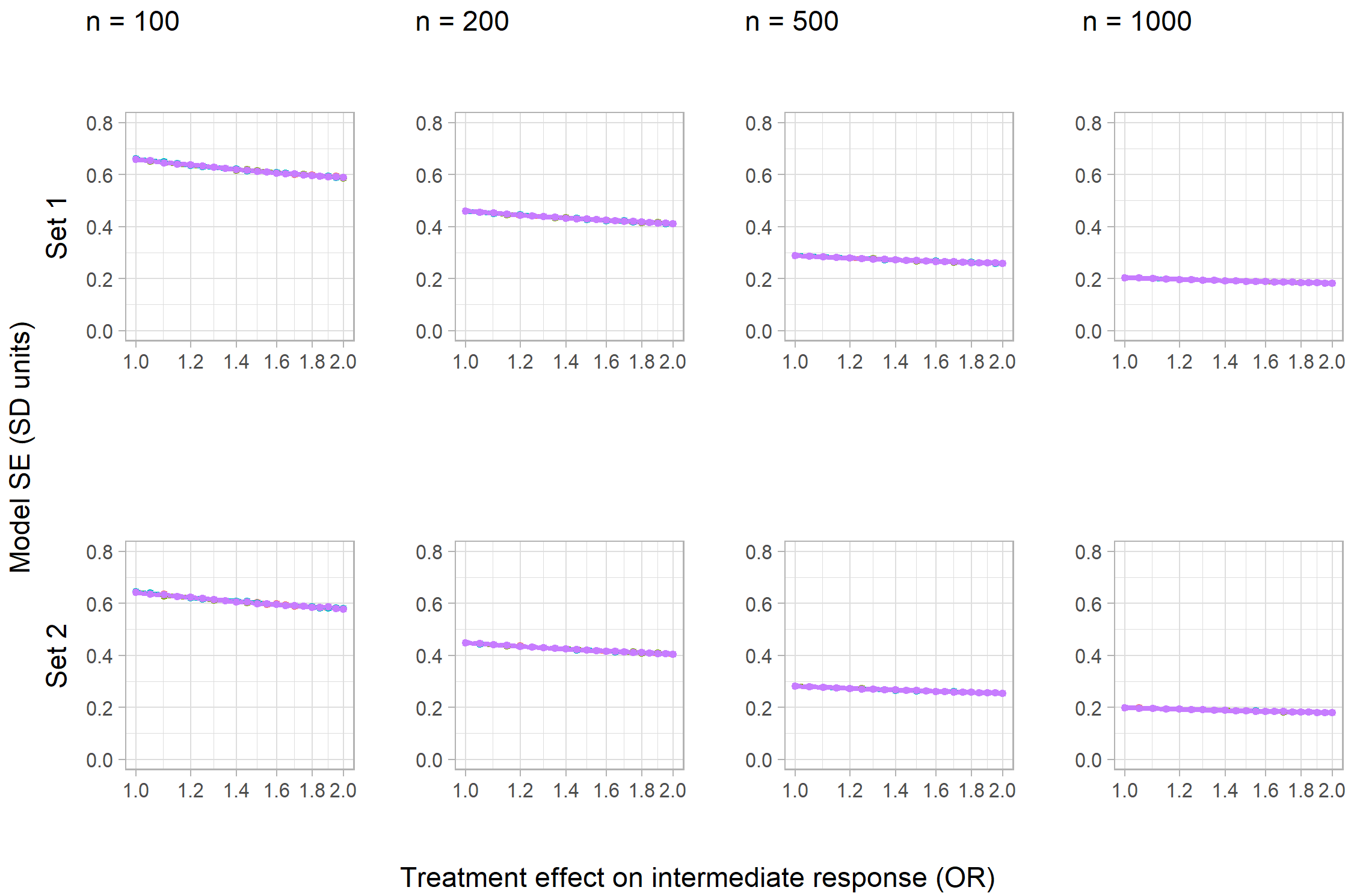


S Figure 15: Model SE of a simple difference in means in the continuous outcome study (sensitivity analysis A) increased confounding). Colour indicates treatment effect on the outcome variable (SDs): red = 0, green = 0.2, blue = 1, purple = 5


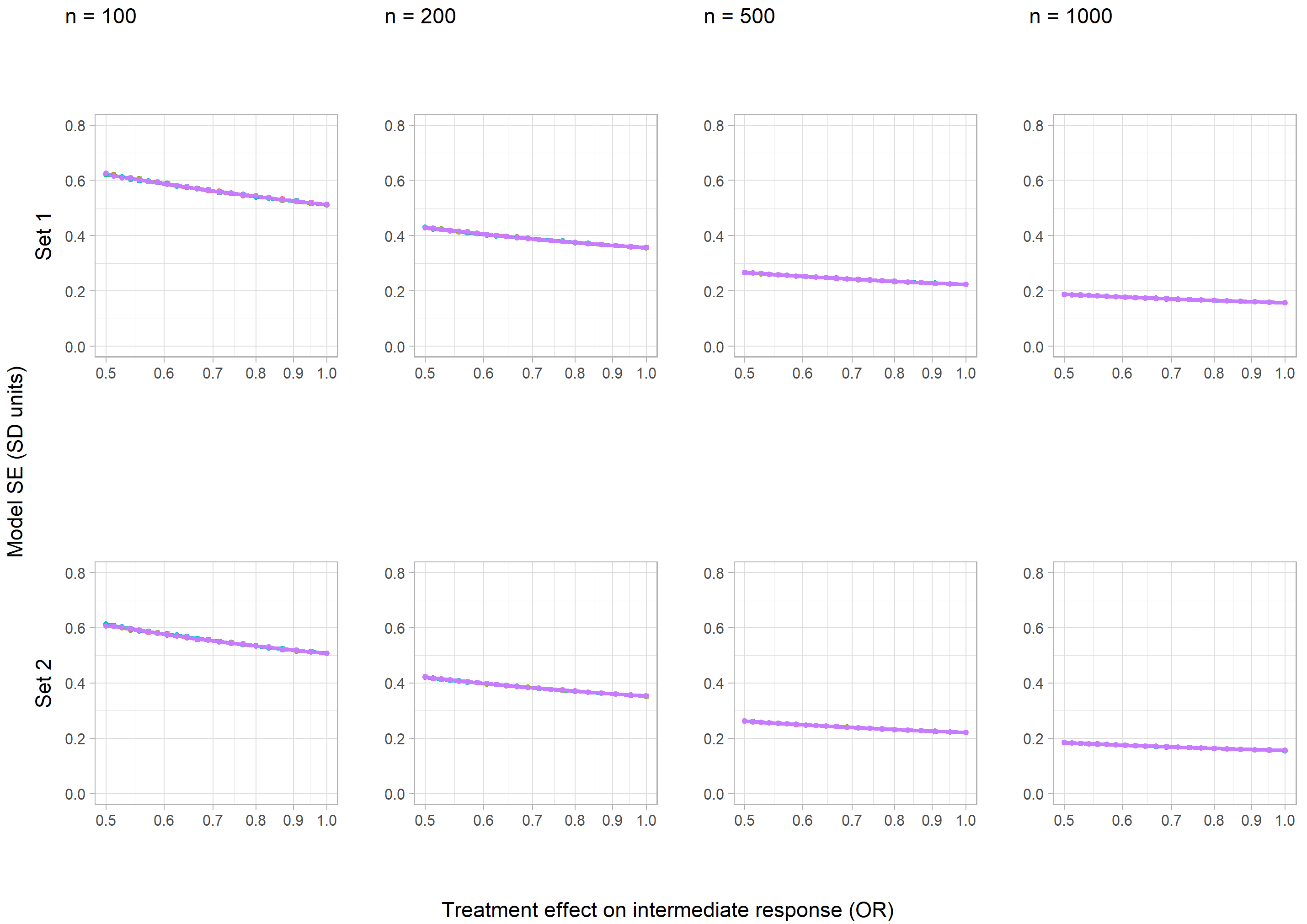


S Figure 16: Model SE of a simple difference in means in the continuous outcome study (sensitivity analysis B), changed direction of effect on intermediate). Colour indicates treatment effect on the outcome variable (SDs): red = 0, green = 0.2, blue = 1, purple = 5.


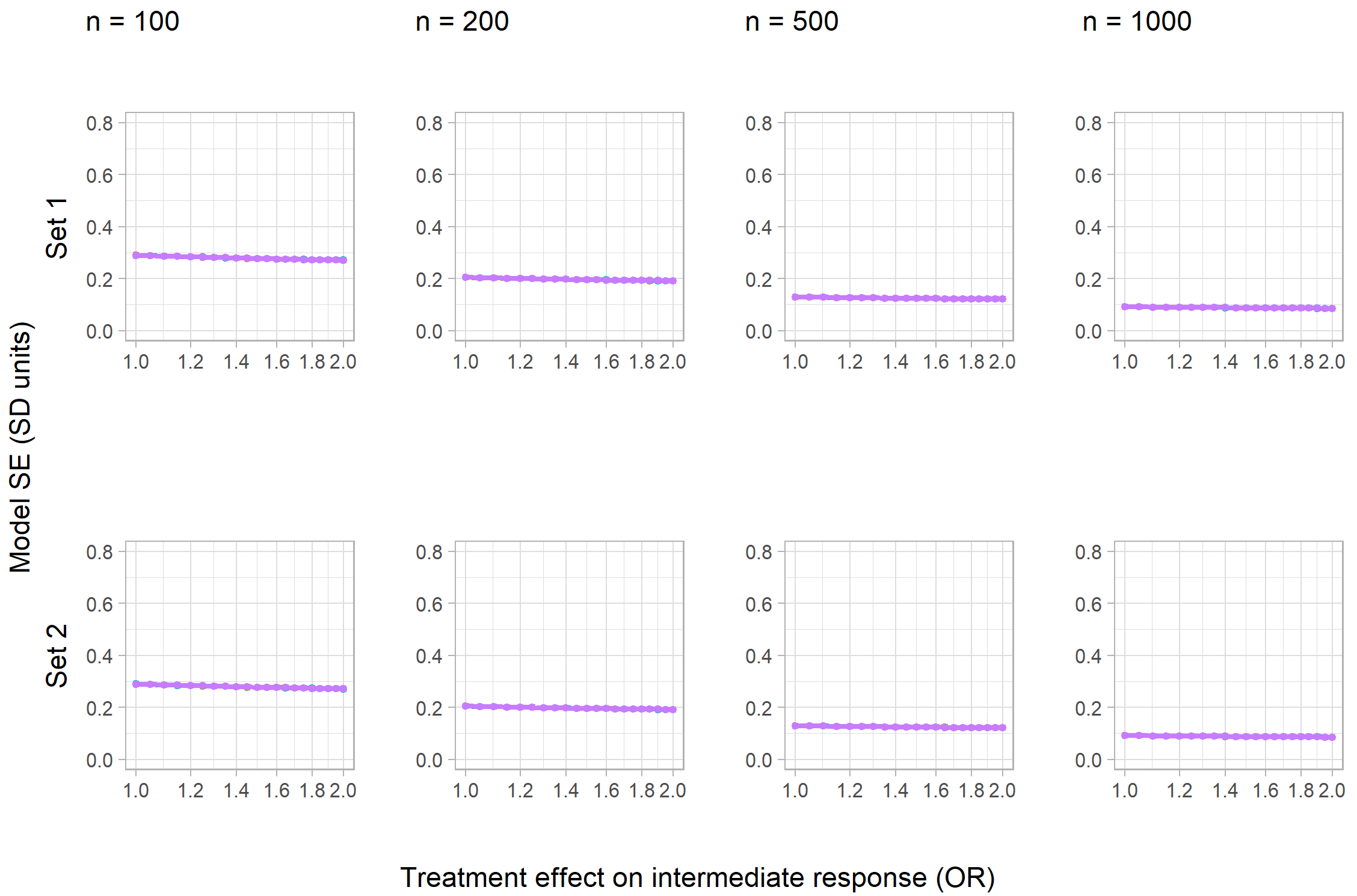


S Figure 17: Model SE of a simple difference in means in the continuous outcome study (sensitivity analysis C) increased event rate). Colour indicates treatment effect on the outcome variable (SDs): red = 0, green = 0.2, blue = 1, purple = 5.

# Binary outcomes

## Missing data


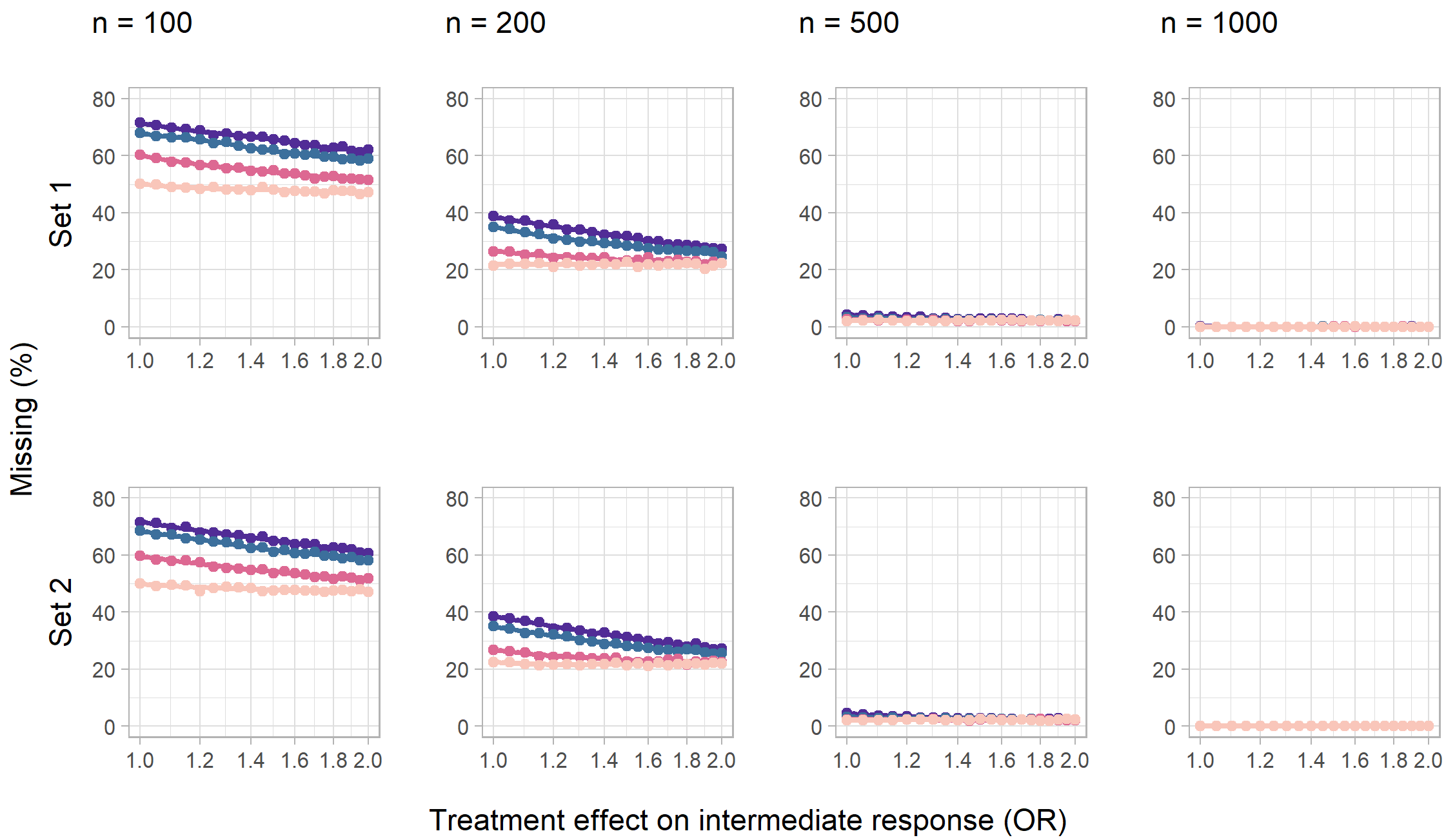


S Figure 18: Amount of missing data due to the treatment effect OR being inestimable in the binary outcome study (core scenarios). Colour indicates treatment effect on the outcome (ORs) (Purple = 1, blue = 1.2, darkpink = 2, light pink = 5)


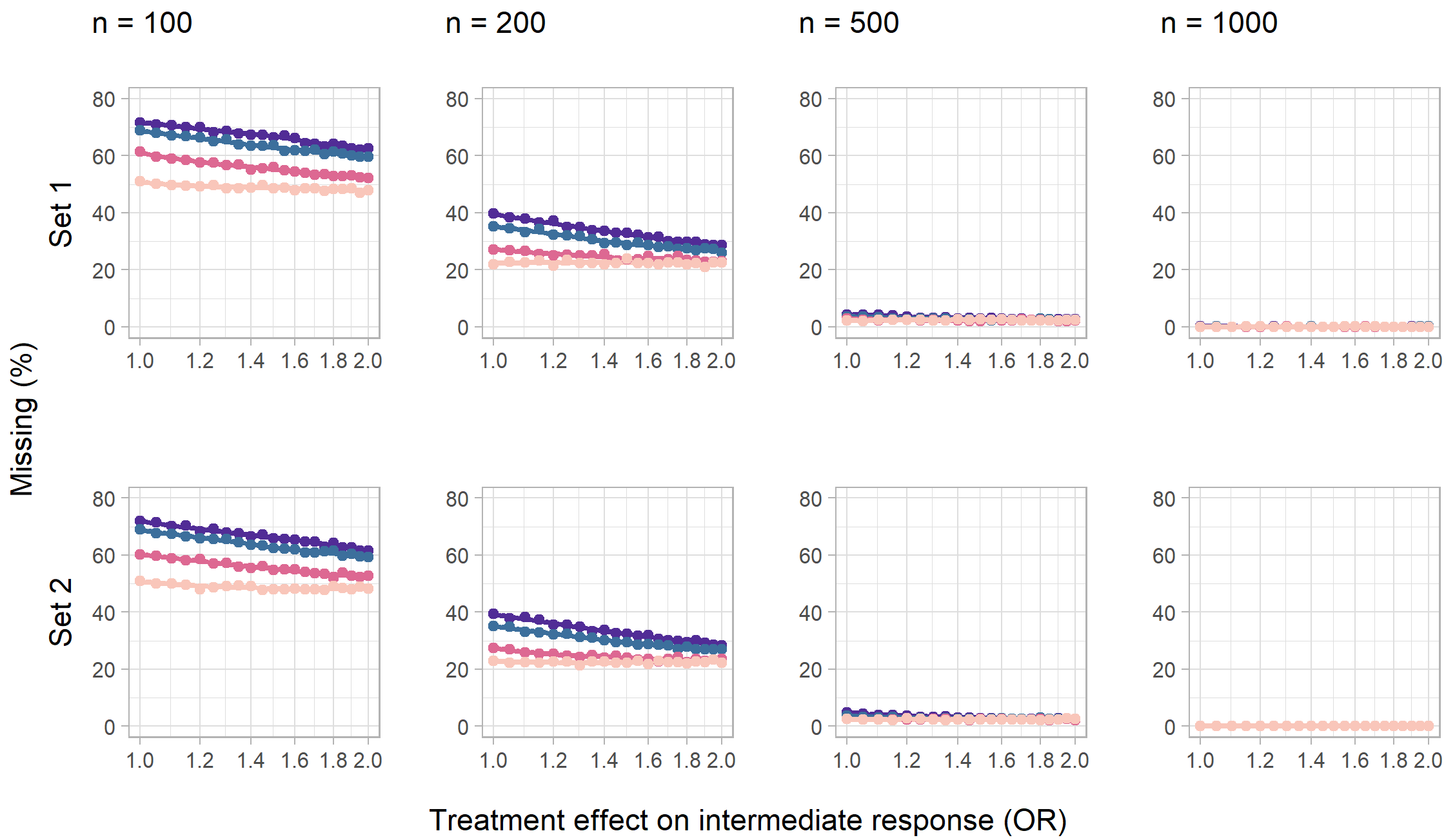


S Figure 19: Amount of missing data due to the treatment effect OR being inestimable in the binary outcome study (sensitivity analysis A) increased confounding). Colour indicates treatment effect on the outcome (ORs) (Purple = 1, blue = 1.2, darkpink = 2, light pink = 5)


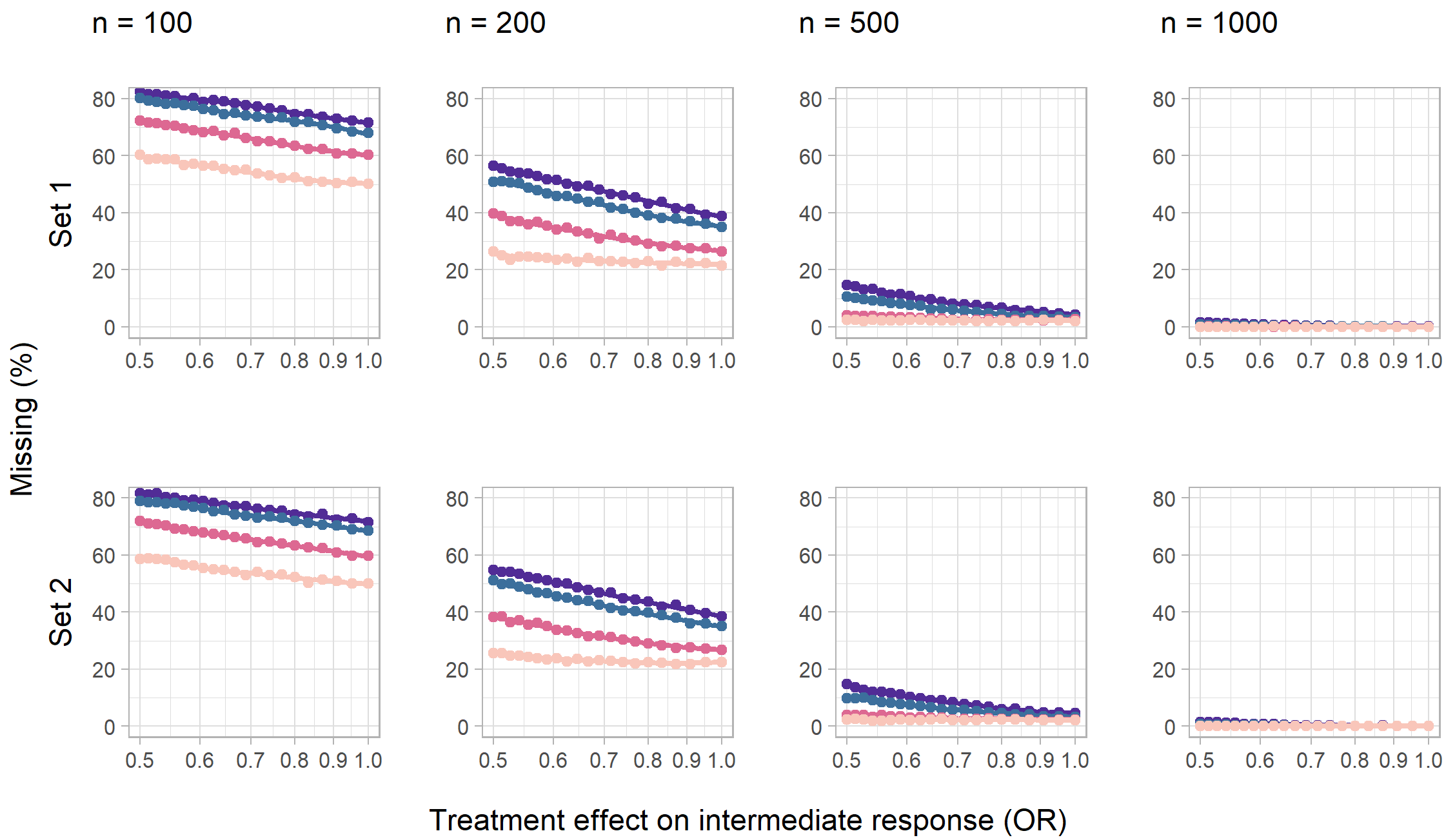


S Figure 20: Amount of missing data due to the treatment effect OR being inestimable in the binary outcome study (sensitivity analysis B) changed sign of treatment effect on intermediate). Colour indicates treatment effect on the outcome (ORs) (Purple = 1, blue = 1.2, darkpink = 2, light pink = 5)


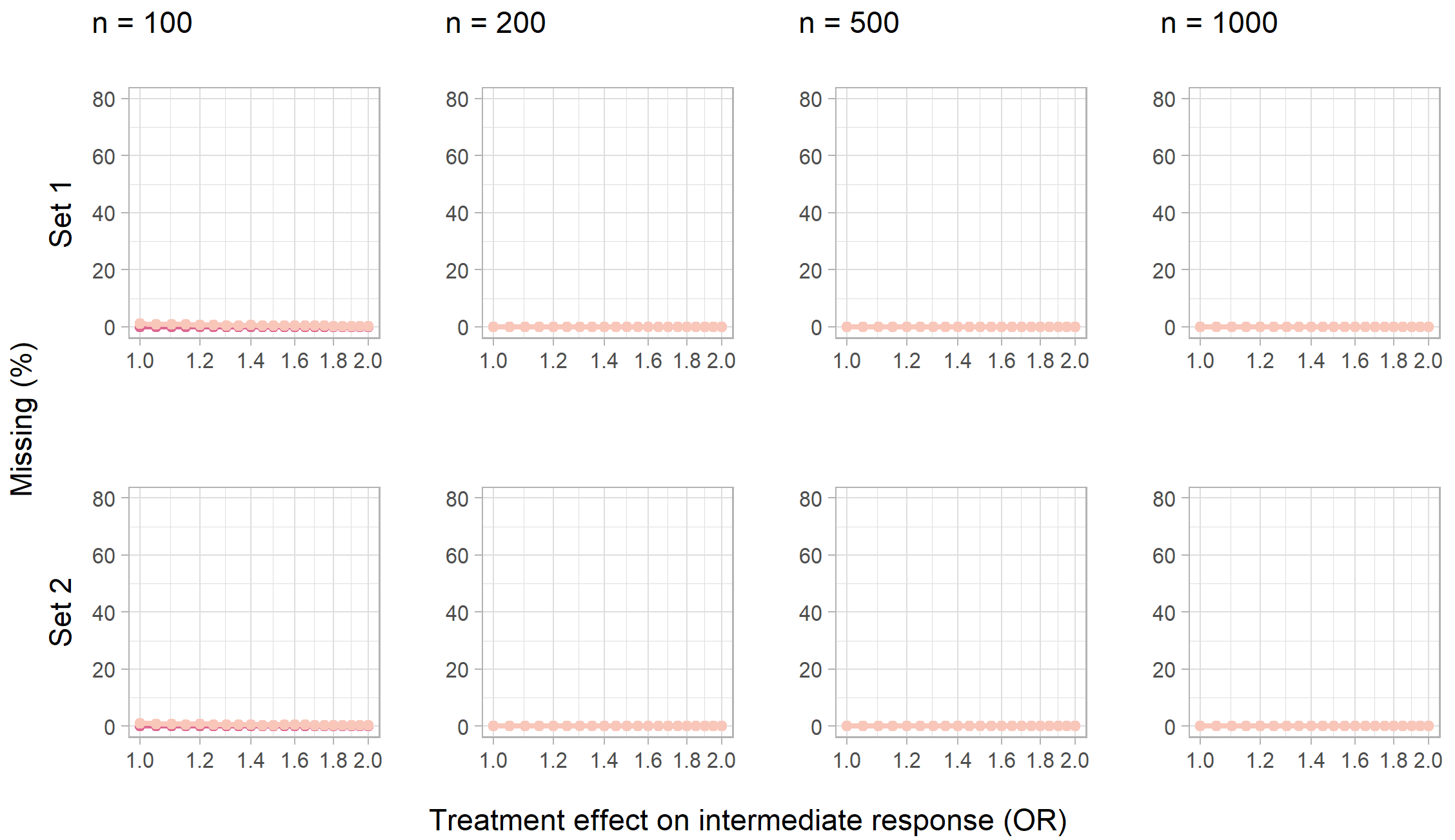


S Figure 21: Amount of missing data due to the treatment effect OR being inestimable in the binary outcome study (sensitivity analysis C) increased event rate). Colour indicates treatment effect on the outcome (ORs) (Purple = 1, blue = 1.2, darkpink = 2, light pink = 5).


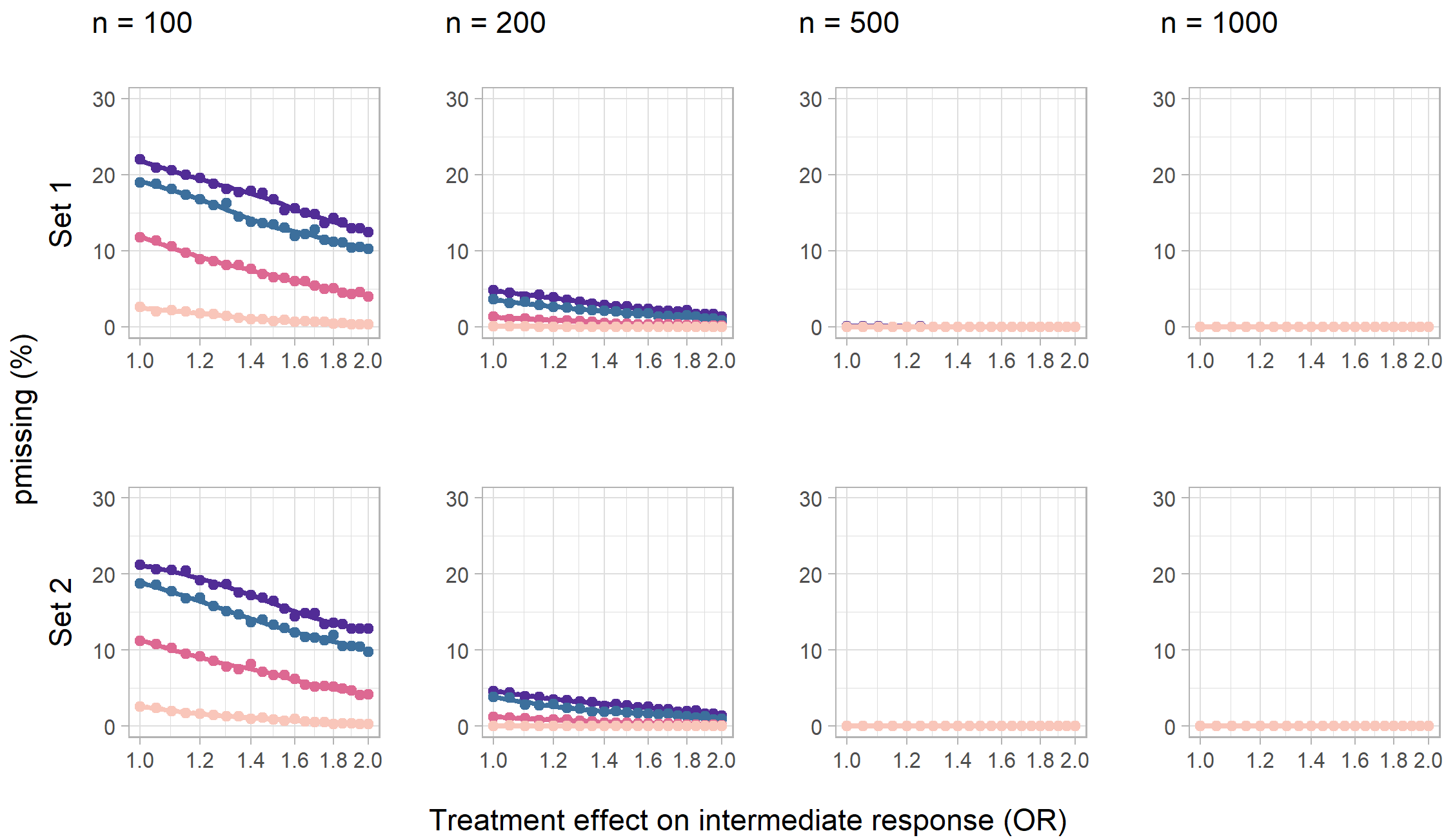


S Figure 22: Amount of missing data due to the chi-squared statistic being incalculable in the binary outcome study (core scenarios). Colour indicates treatment effect on the outcome (ORs) (Purple = 1, blue = 1.2, darkpink = 2, light pink = 5)


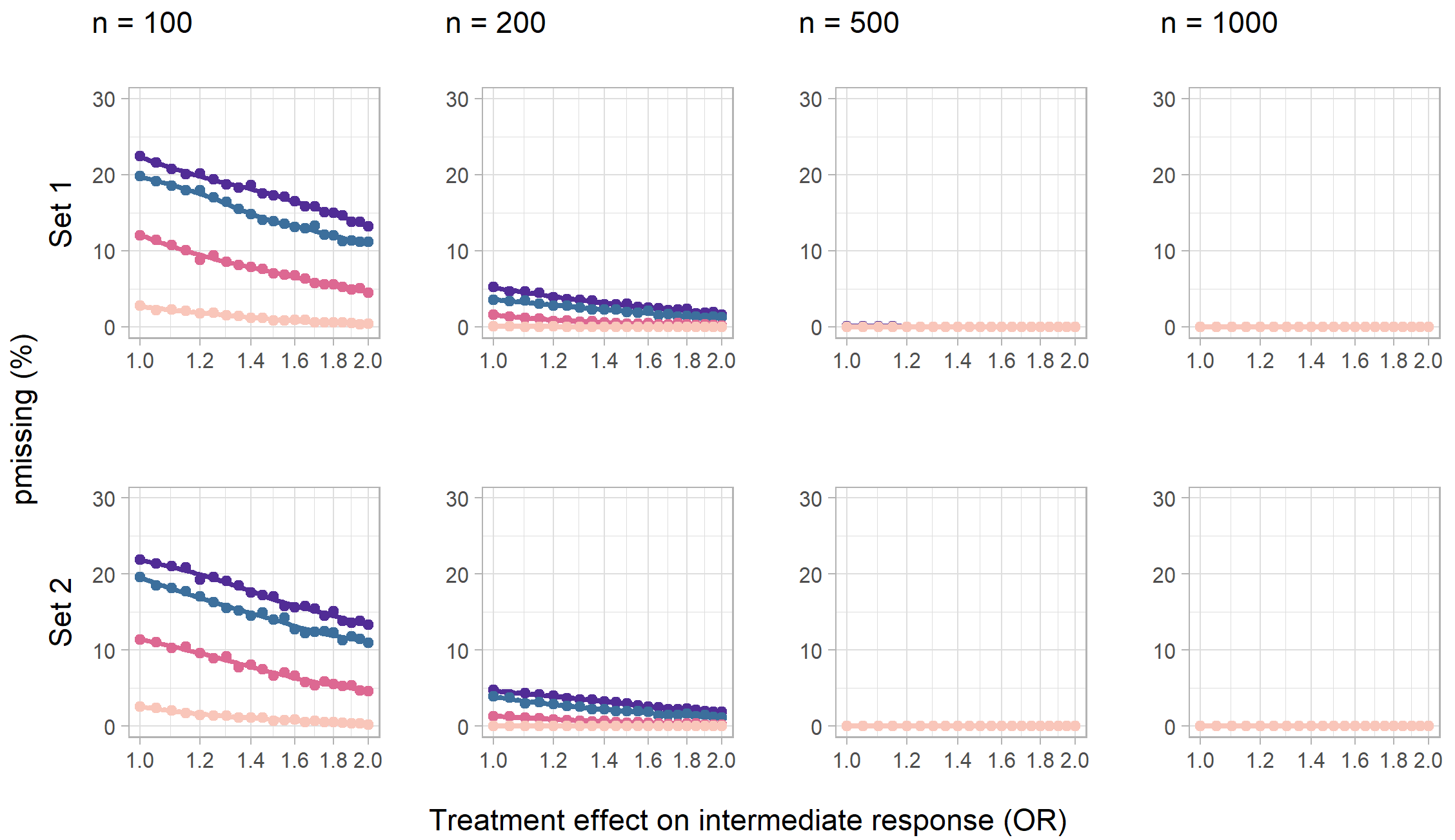


S Figure 23: Amount of missing data due to the chi-squared statistic being incalculable in the binary outcome study (sensitivity analysis A) increased confounding). Colour indicates treatment effect on the outcome (ORs) (Purple = 1, blue = 1.2, darkpink = 2, light pink = 5).


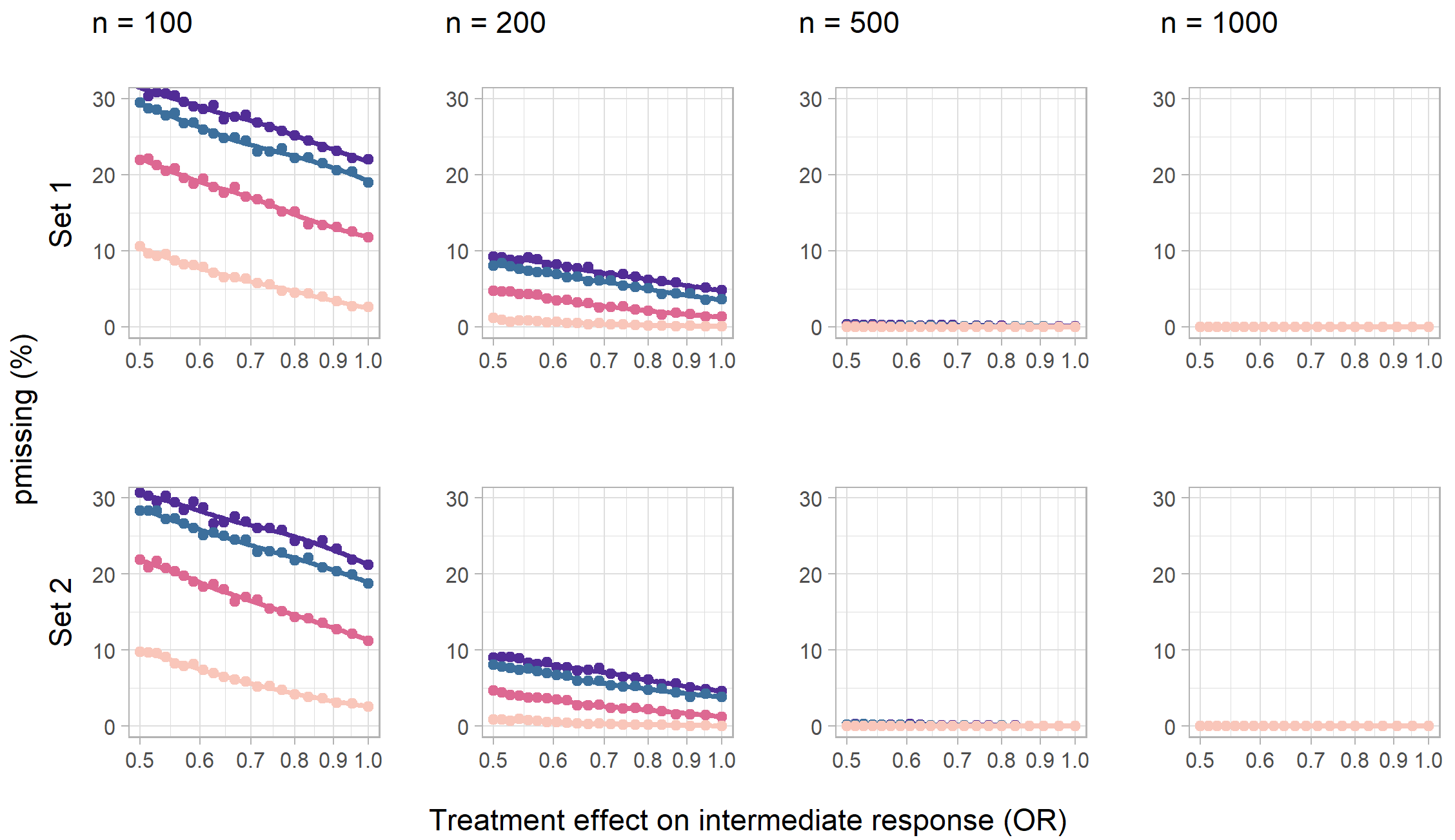


S Figure 24: Amount of missing data due to the chi-squared statistic being incalculable in the binary outcome study (sensitivity analysis B) changed direction of treatment effect on intermediate). Colour indicates treatment effect on the outcome (ORs) (Purple = 1, blue = 1.2, darkpink = 2, light pink = 5)


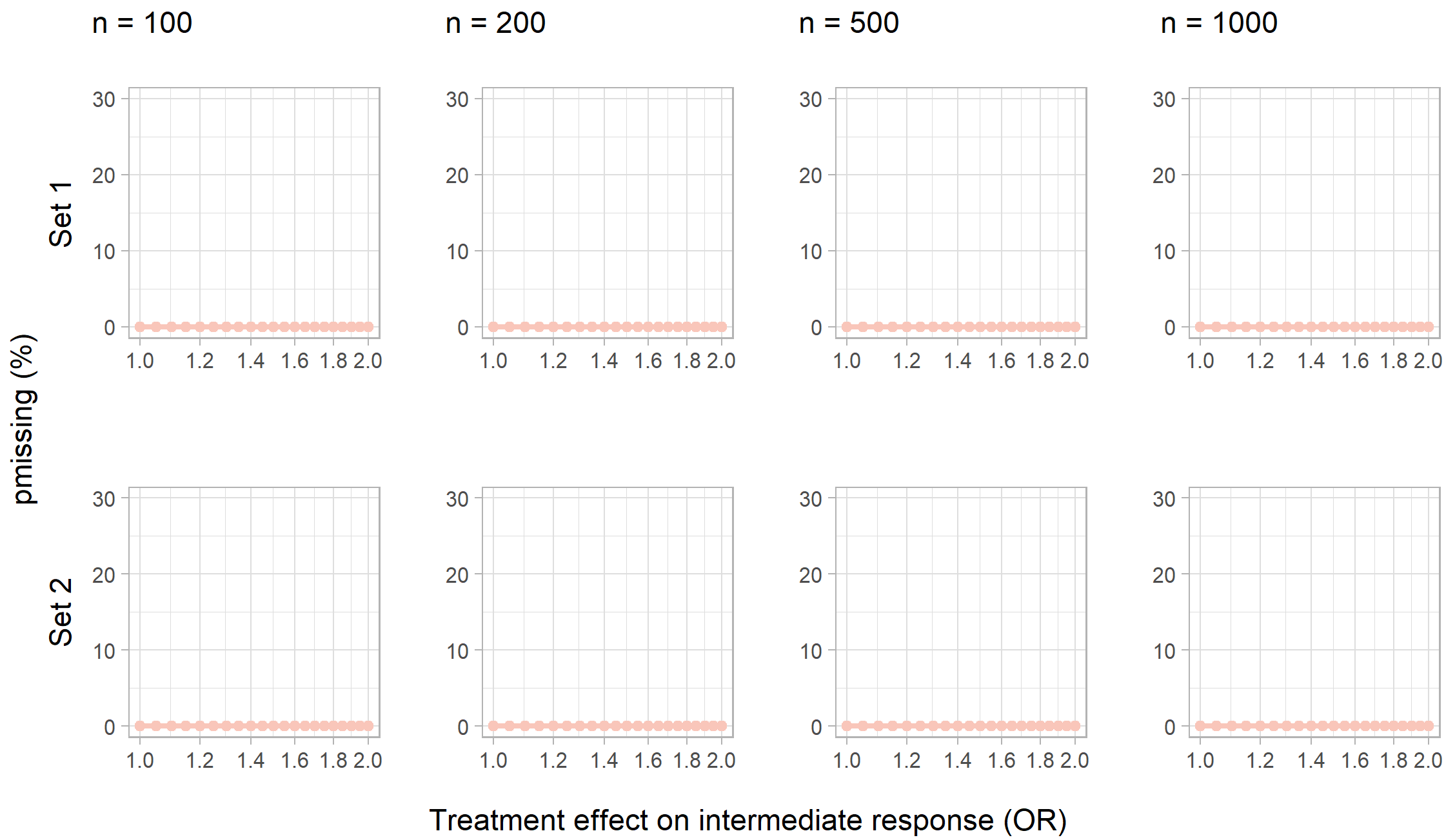


S Figure 25: Amount of missing data due to the chi-squared statistic being incalculable in the binary outcome study (sensitivity analysis C) increased event rate). Colour indicates treatment effect on the outcome (ORs) (Purple = 1, blue = 1.2, darkpink = 2, light pink = 5)

## Bias


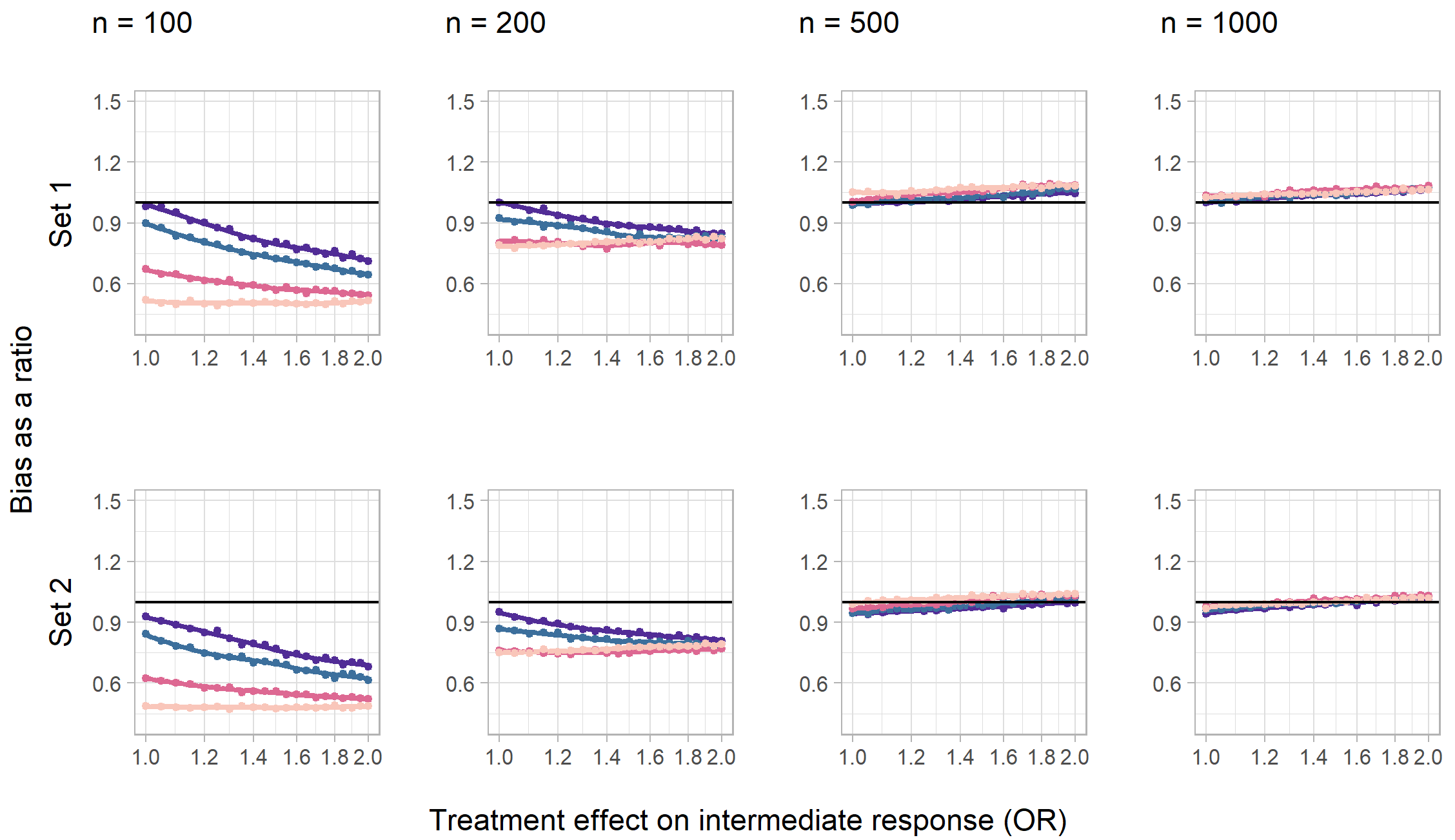


S Figure 26: Bias expressed as a ratio of estimated to true odds ratio (ROR) in the binary outcome study (sensitivity analysis A), increased confounding). Colour indicates treatment effect on the outcome (ORs) (Purple = 1, blue = 1.2, darkpink = 2, light pink = 5)


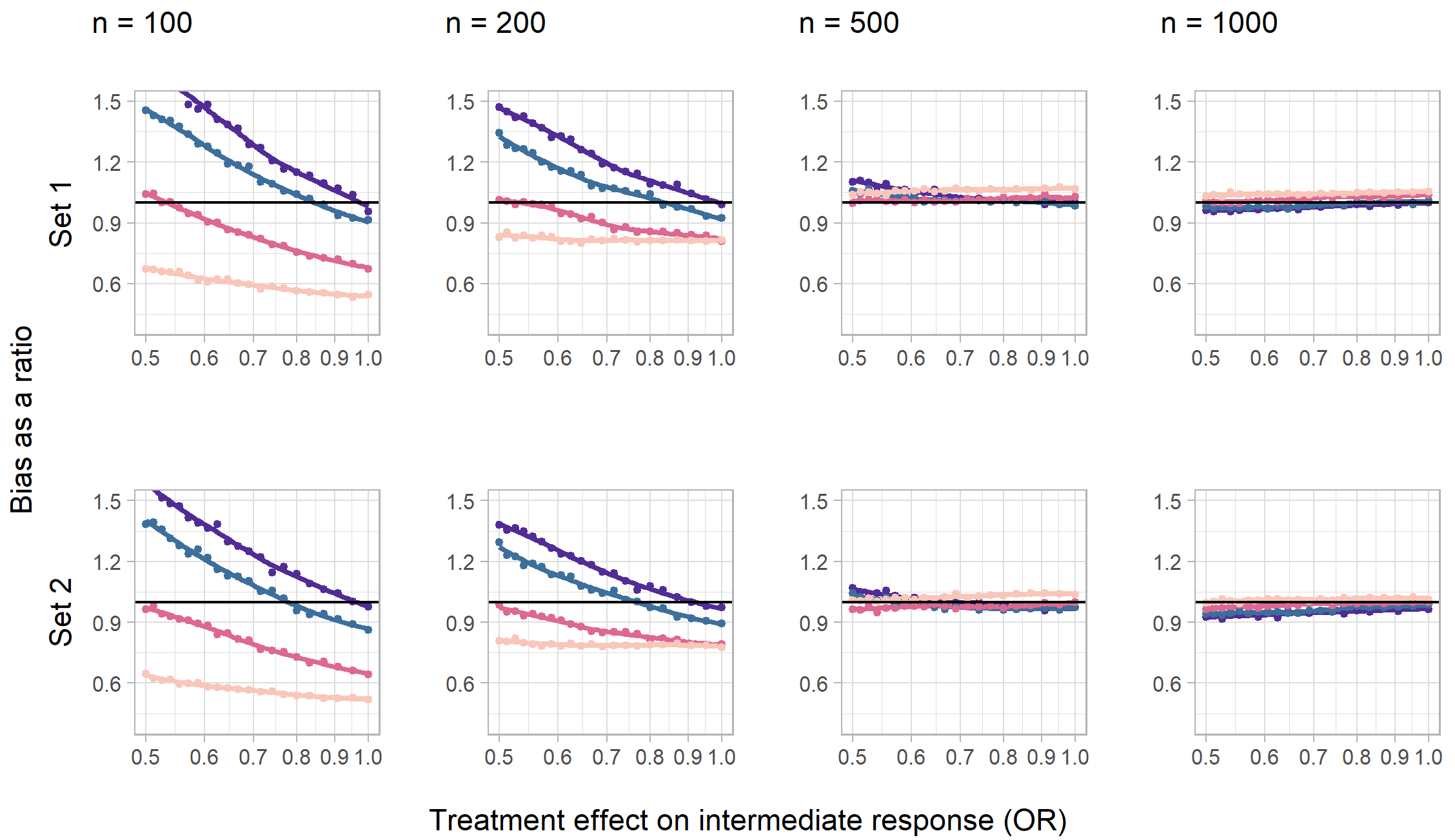


S Figure 27: Bias expressed as a ratio of estimated to true odds ratio (ROR) in the binary outcome study (sensitivity analysis B), changed direction of treatment effect on intermediate). Colour indicates treatment effect on the outcome (ORs) (Purple = 1, blue = 1.2, darkpink = 2, light pink = 5)


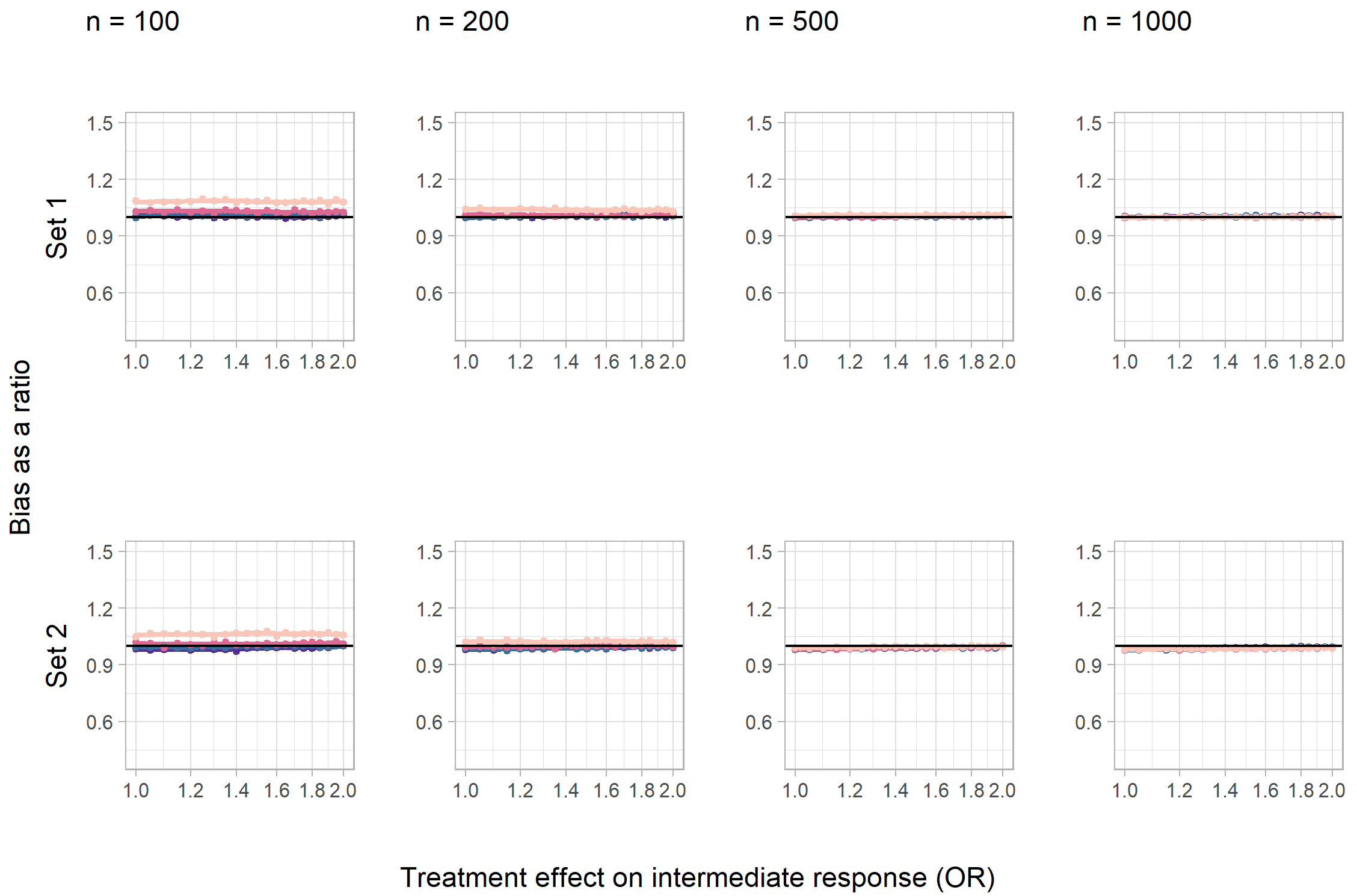


S Figure 28: Bias expressed as a ratio of estimated to true odds ratio (ROR) in the binary outcome study (sensitivity analysis C), increased event rate). Colour indicates treatment effect on the outcome (ORs) (Purple = 1, blue = 1.2, darkpink = 2, light pink = 5)

## Coverage


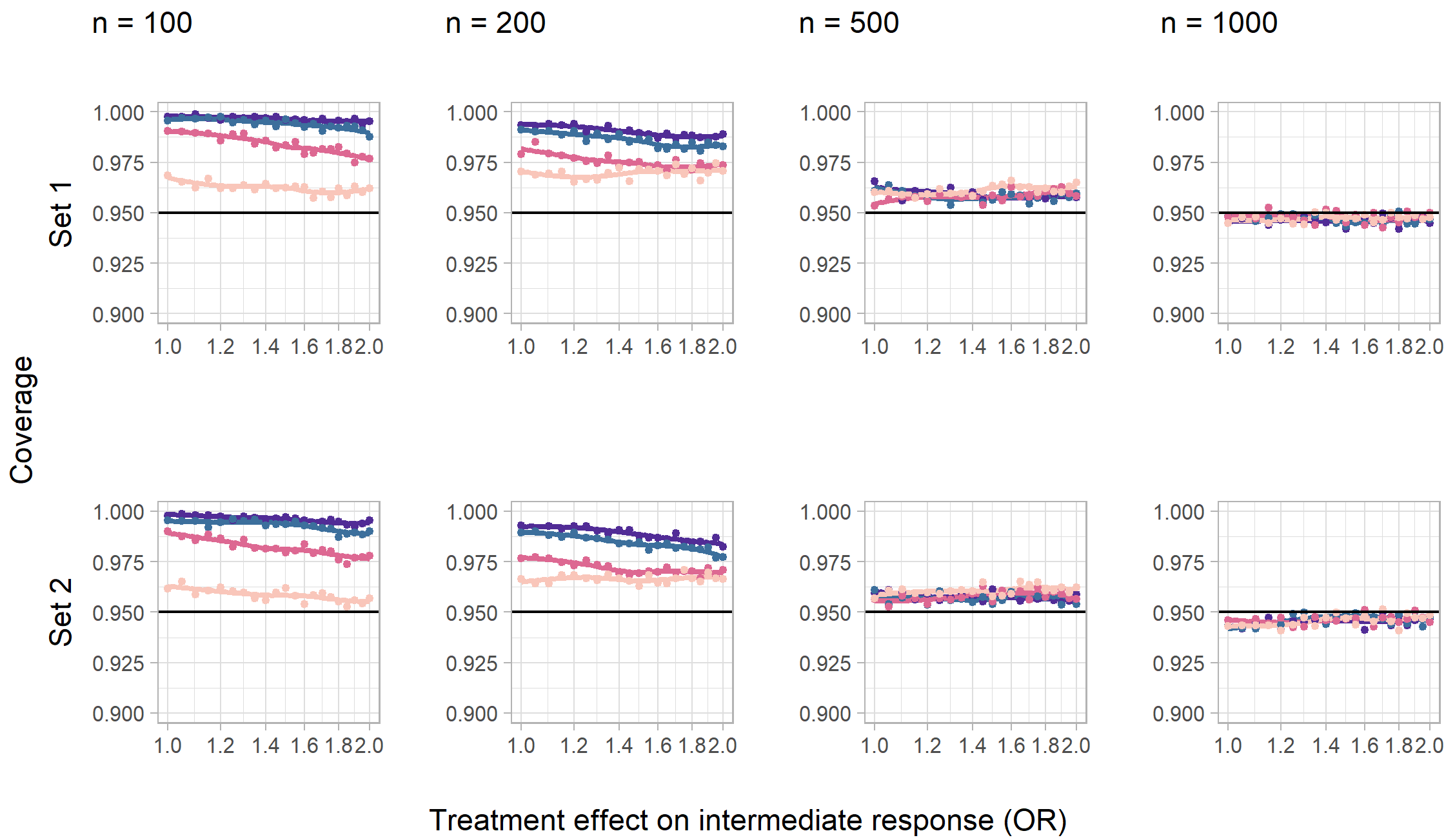


S Figure 29: Coverage of 95% confidence interval obtained using logistic regression in the binary outcome study (sensitivity analysis A) increased confounding). Colour indicates treatment effect on the outcome (ORs) (Purple = 1, blue = 1.2, darkpink = 2, light pink = 5).


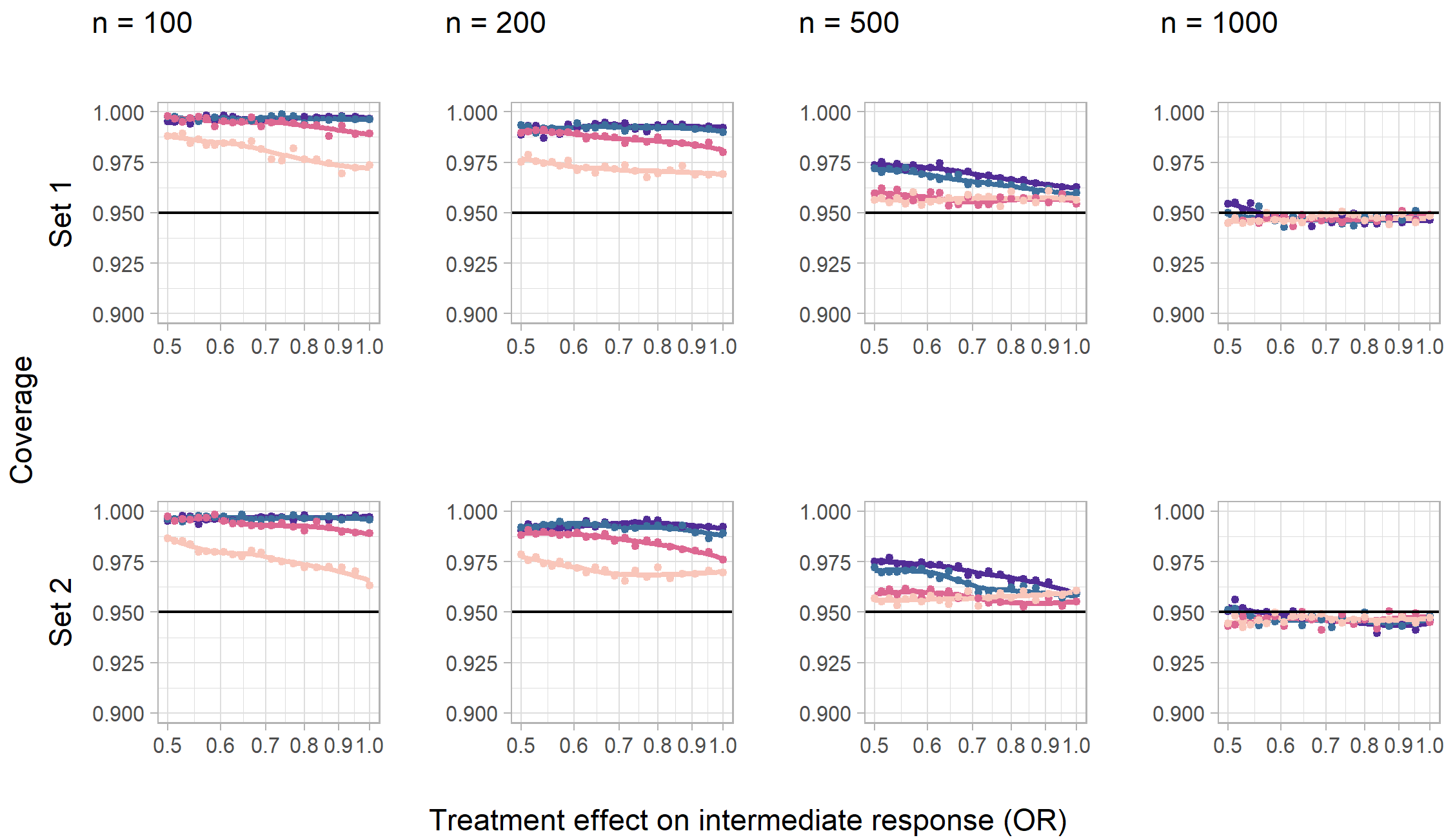


S Figure 30: Coverage of 95% confidence interval obtained using logistic regression in the binary outcome study (sensitivity analysis B) changed direction of treatment effect on intermediate). Colour indicates treatment effect on the outcome (ORs) (Purple = 1, blue = 1.2, darkpink = 2, light pink = 5).


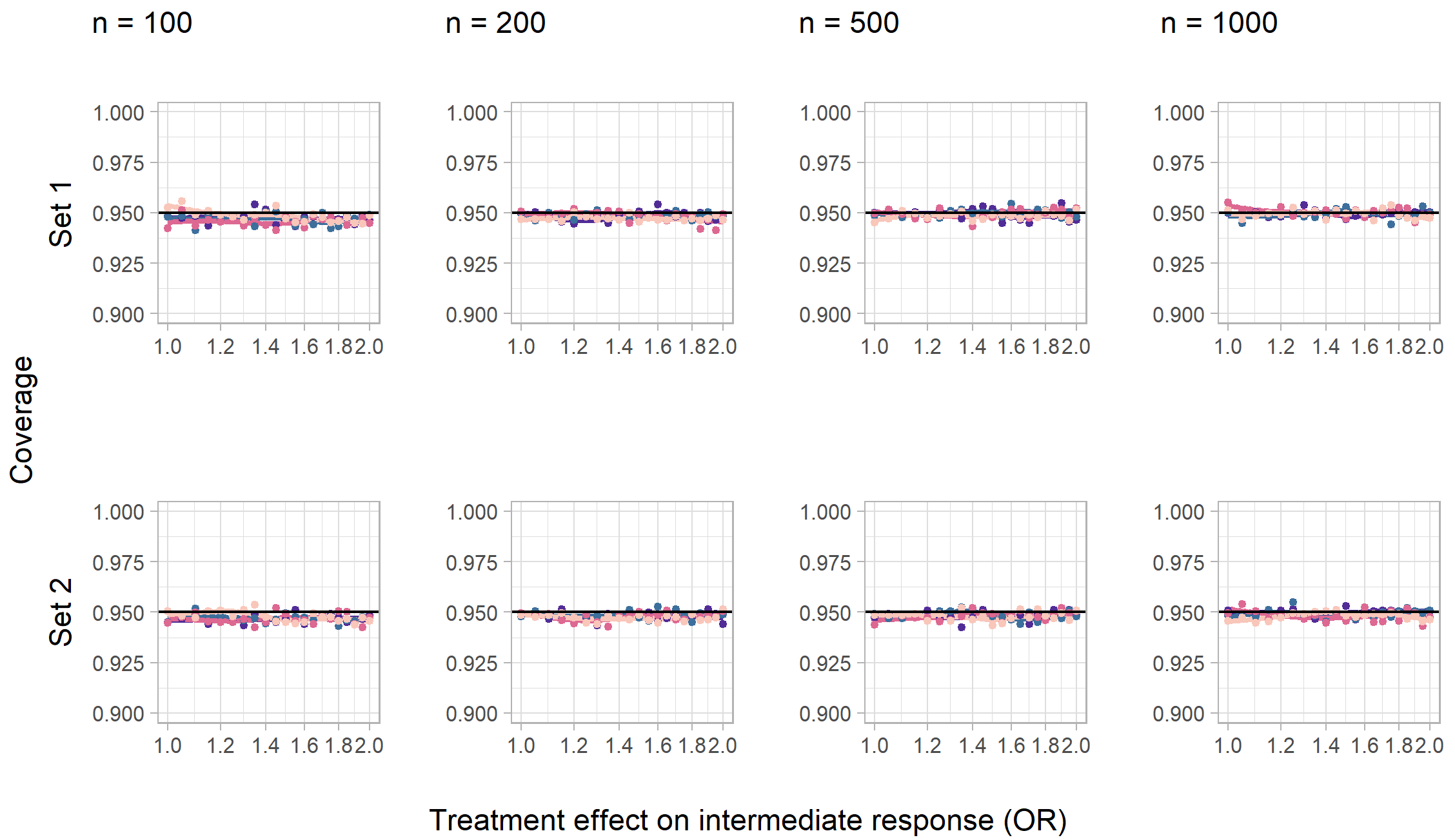


S Figure 31: Coverage of 95% confidence interval obtained using logistic regression in the binary outcome study (sensitivity analysis C) increased event rate). Colour indicates treatment effect on the outcome (ORs) (Purple = 1, blue = 1.2, darkpink = 2, light pink = 5).

## Empirical SE


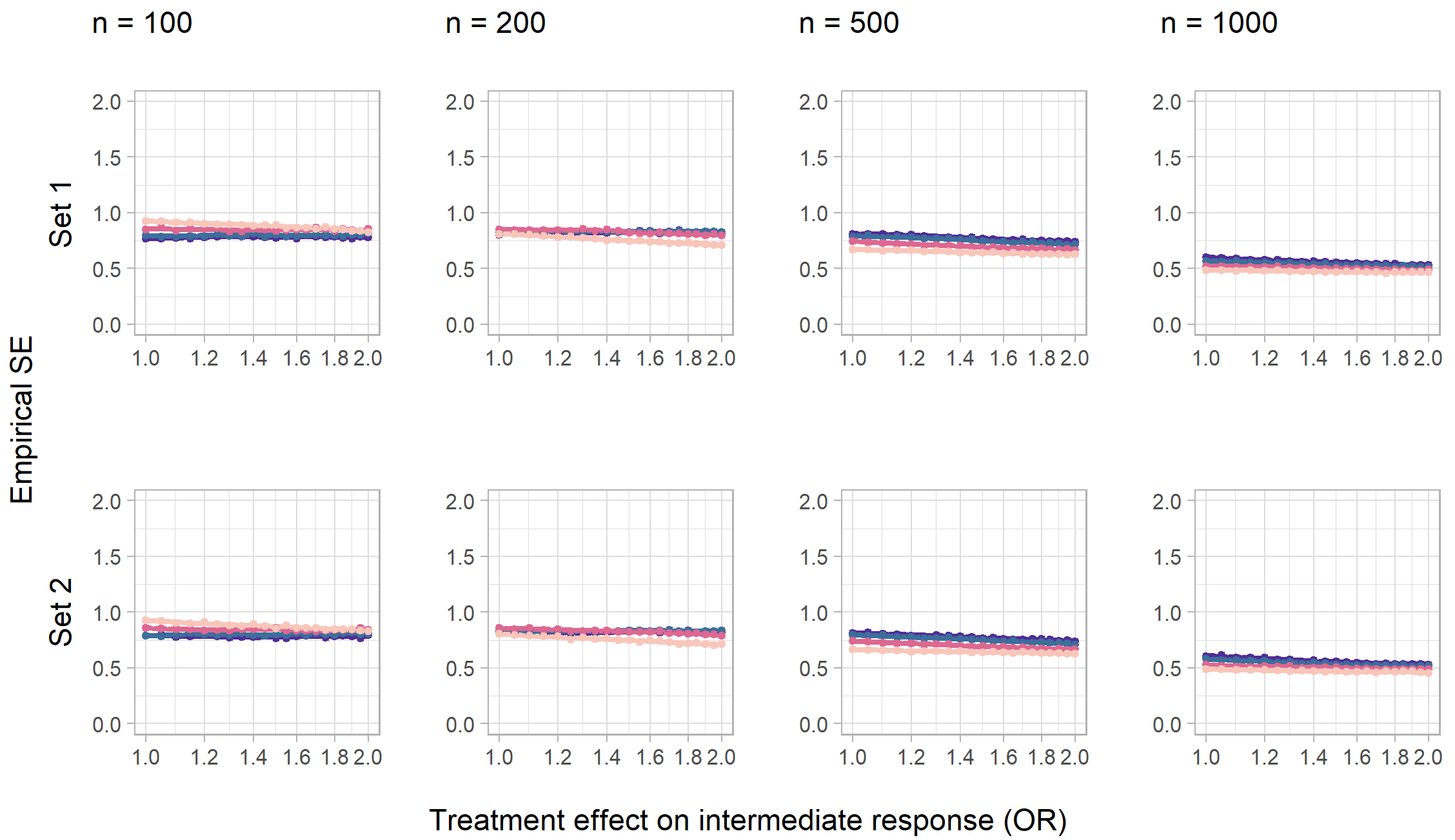


S Figure 32: Empirical SE in the binary outcome study (core scenarios). Colour indicates treatment effect on the outcome (ORs) (Purple = 1, blue = 1.2, darkpink = 2, light pink = 5).


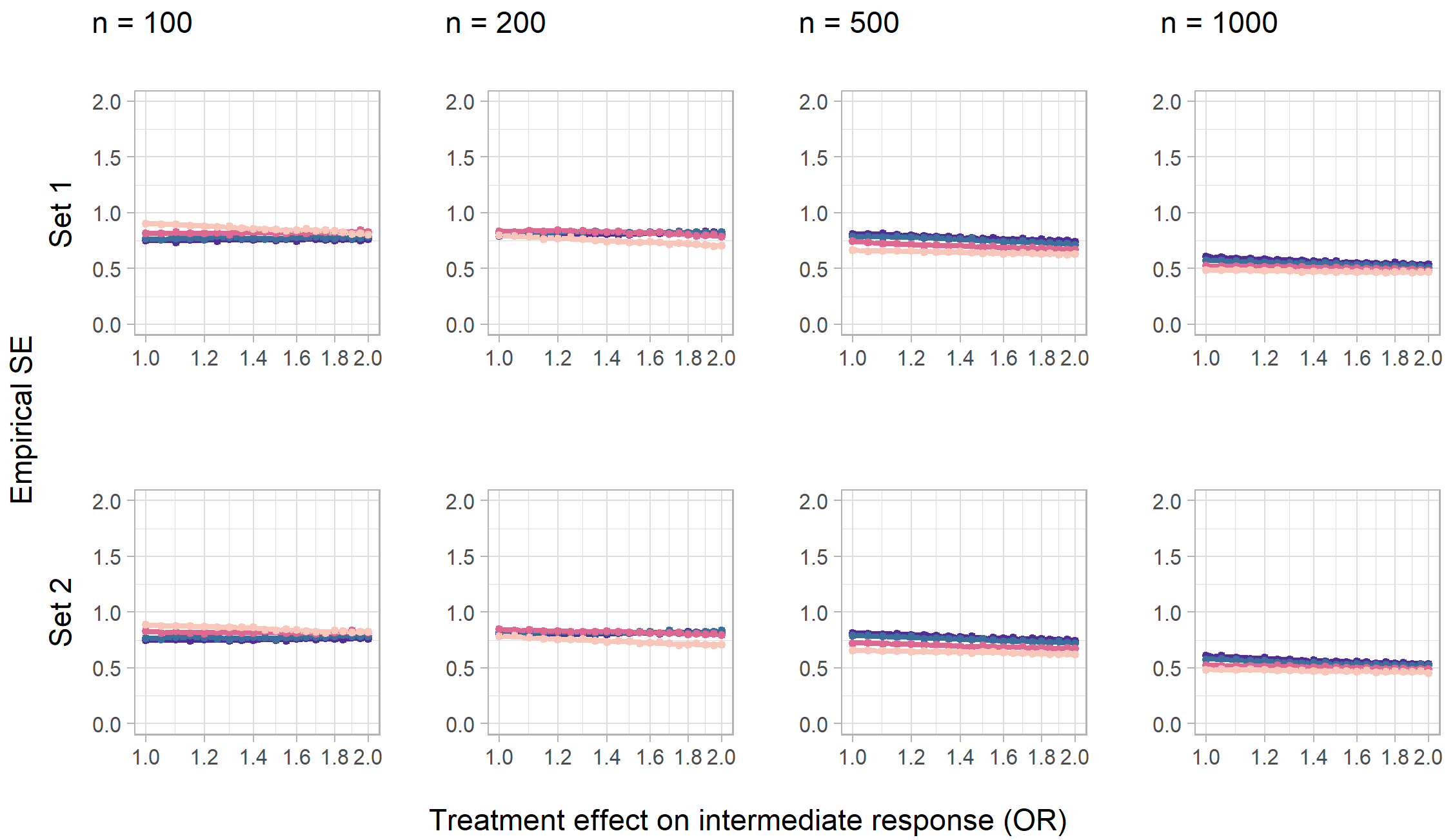


S Figure 33: Empirical SE in the binary outcome study (sensitivity analysis A) increased confounding). Colour indicates treatment effect on the outcome (ORs) (Purple = 1, blue = 1.2, darkpink = 2, light pink = 5).


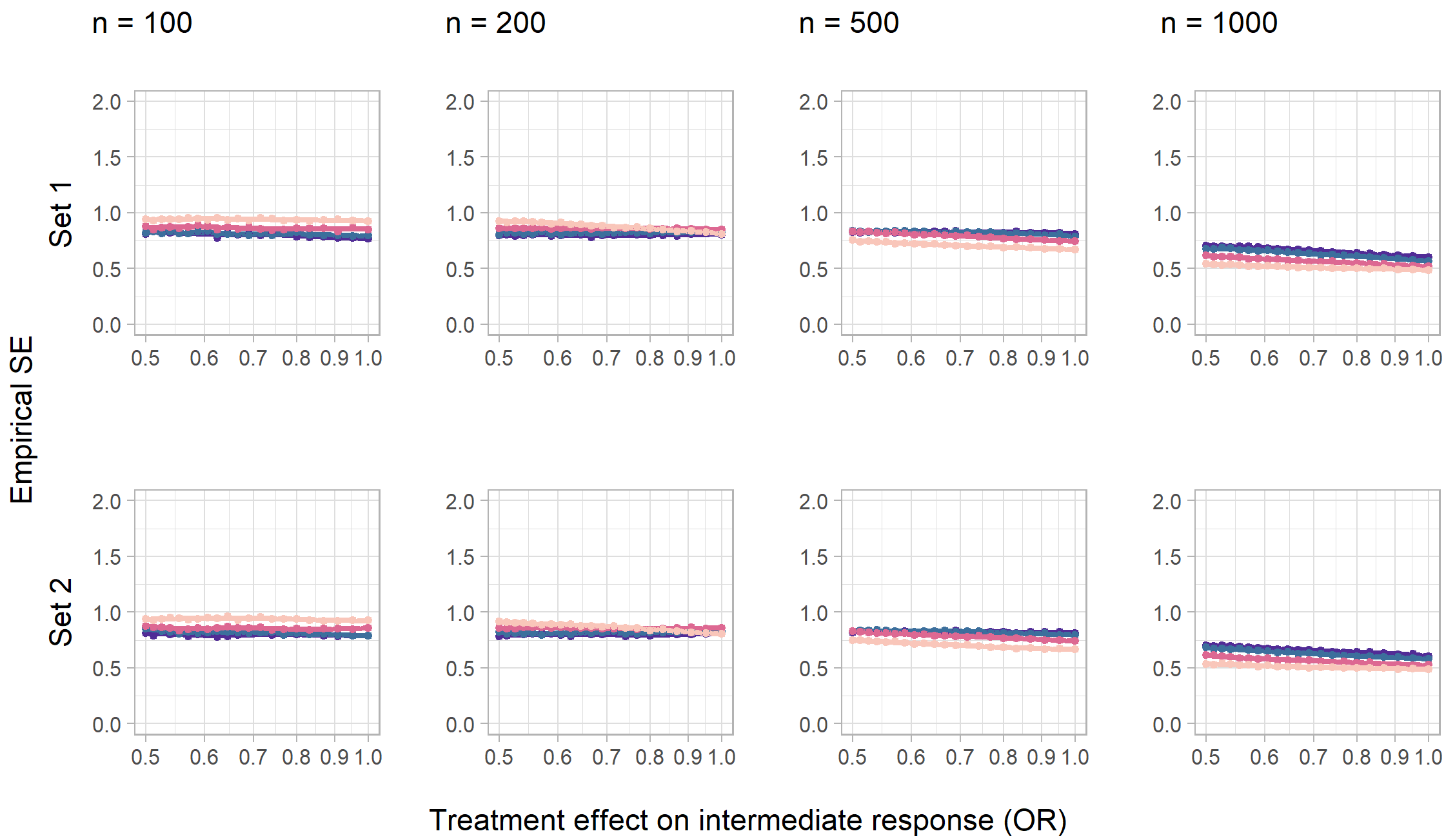


S Figure 34: Empirical SE in the binary outcome study (sensitivity analysis B) changed sign of treatment effect on intermediate variable). Colour indicates treatment effect on the outcome (ORs) (Purple = 1, blue = 1.2, darkpink = 2, light pink = 5).


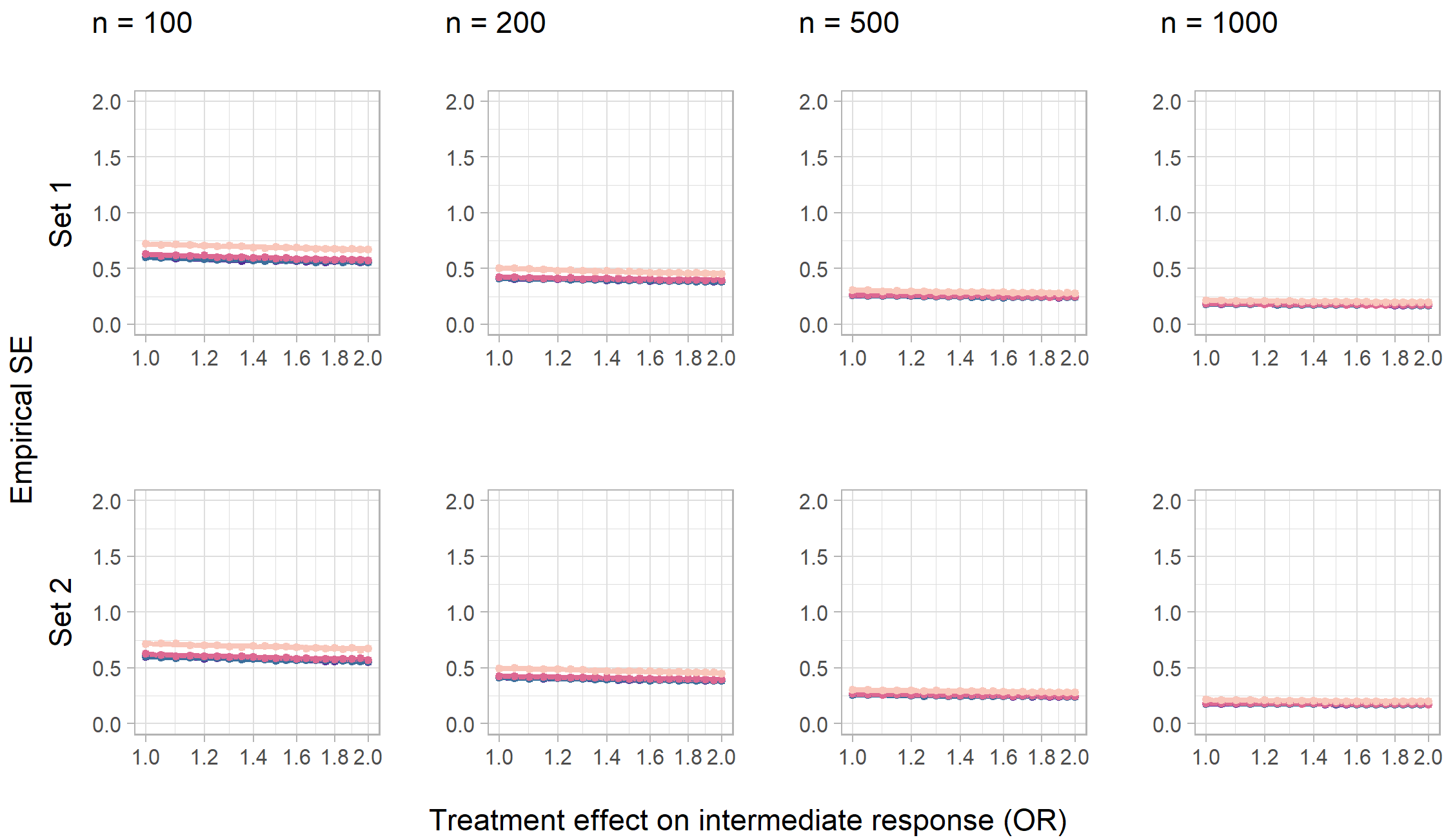


S Figure 35: Empirical SE in the binary outcome study (sensitivity analysis C) increased event rate). Colour indicates treatment effect on the outcome (ORs) (Purple = 1, blue = 1.2, darkpink = 2, light pink = 5).

## Model SE


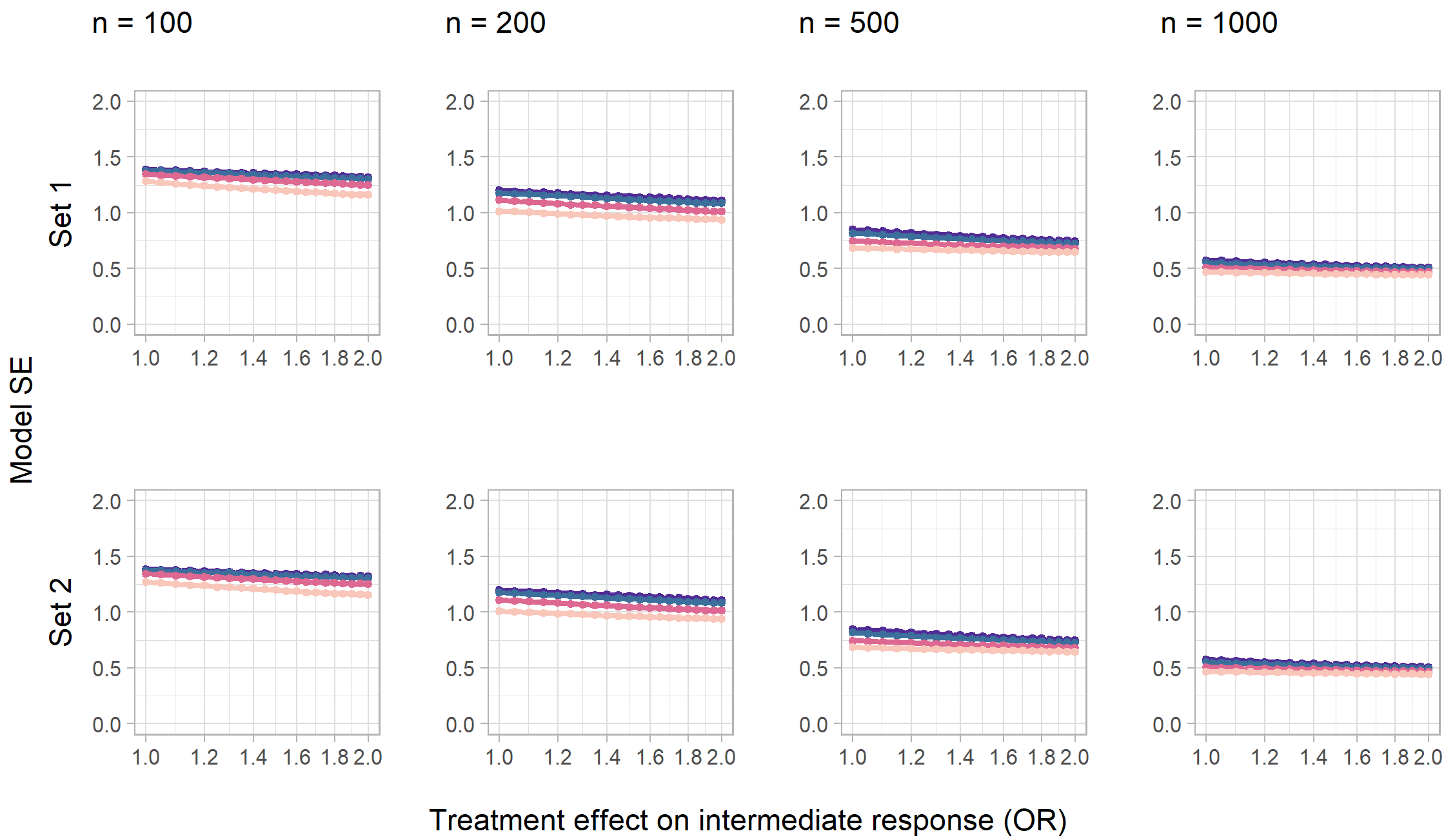


S Figure 36: Model SE in the binary outcome study (core scenarios). Colour indicates treatment effect on the outcome (ORs) (Purple = 1, blue = 1.2, darkpink = 2, light pink = 5).


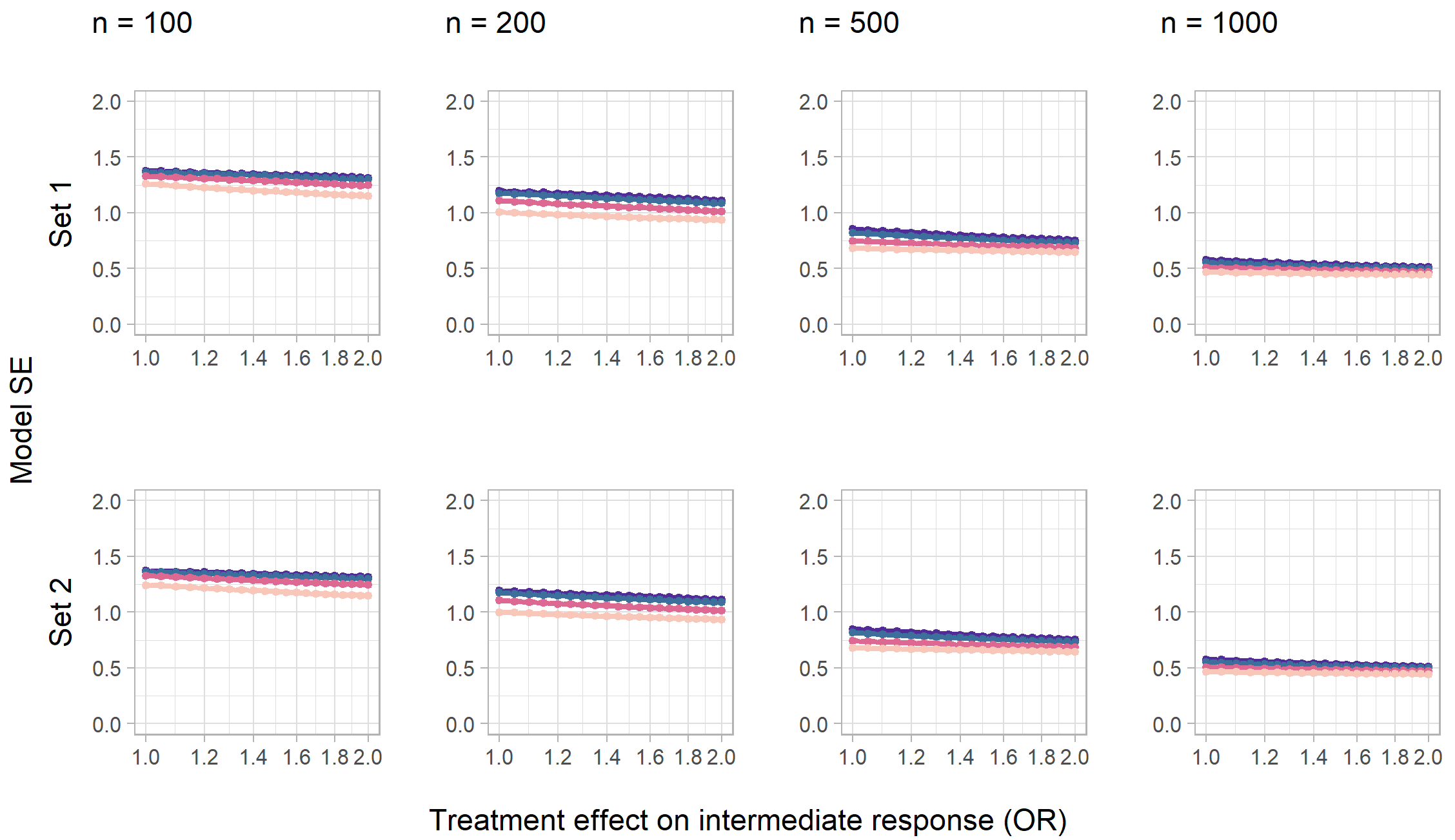


S Figure 37: Model SE in the binary outcome study (sensitivity analysis A) increased confounding). Colour indicates treatment effect on the outcome (ORs) (Purple = 1, blue = 1.2, darkpink = 2, light pink = 5).


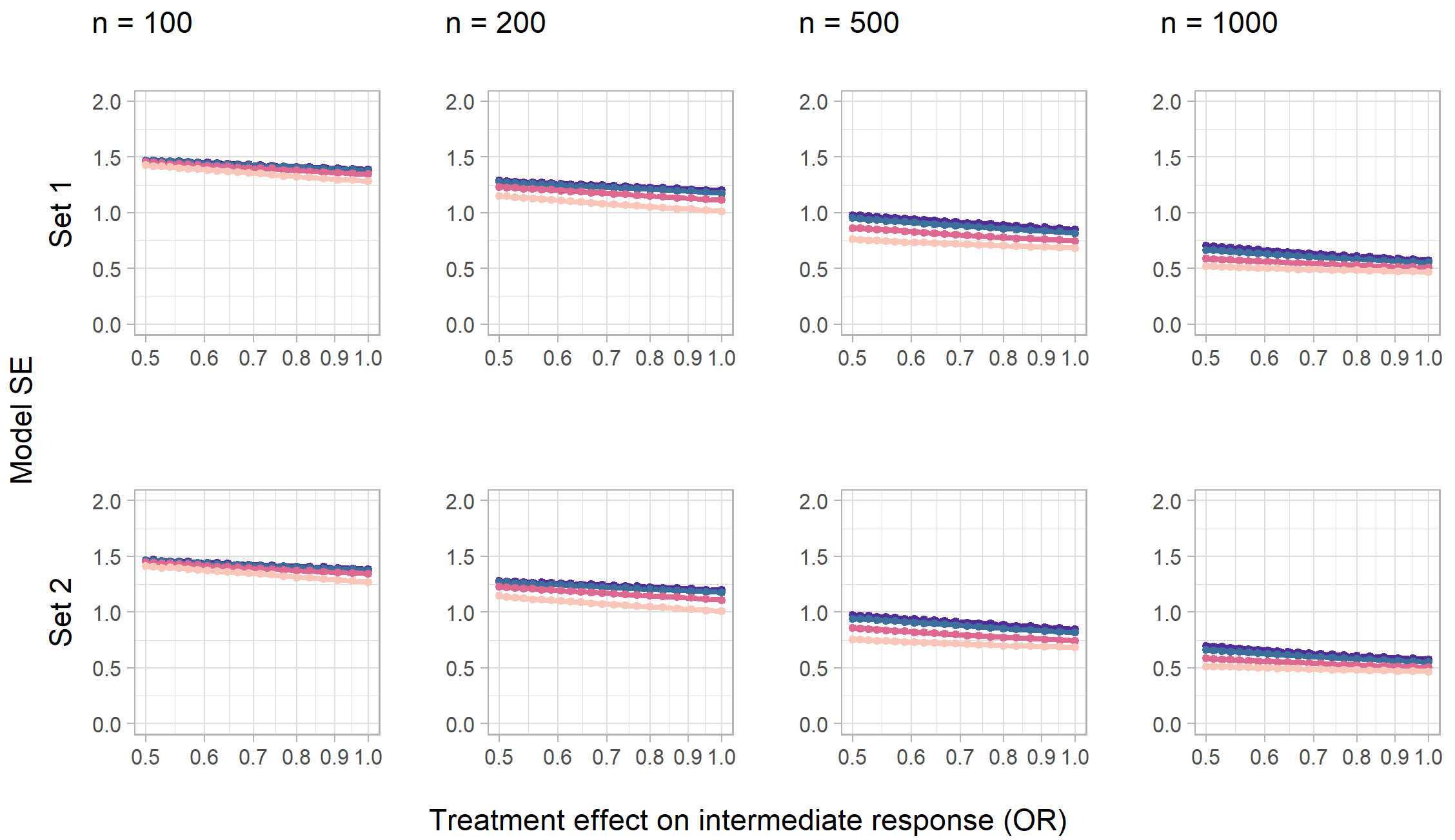


S Figure 38: Model SE in the binary outcome study (sensitivity analysis B) changed sign of effect on intermediate variable). Colour indicates treatment effect on the outcome (ORs) (Purple = 1, blue = 1.2, darkpink = 2, light pink = 5).


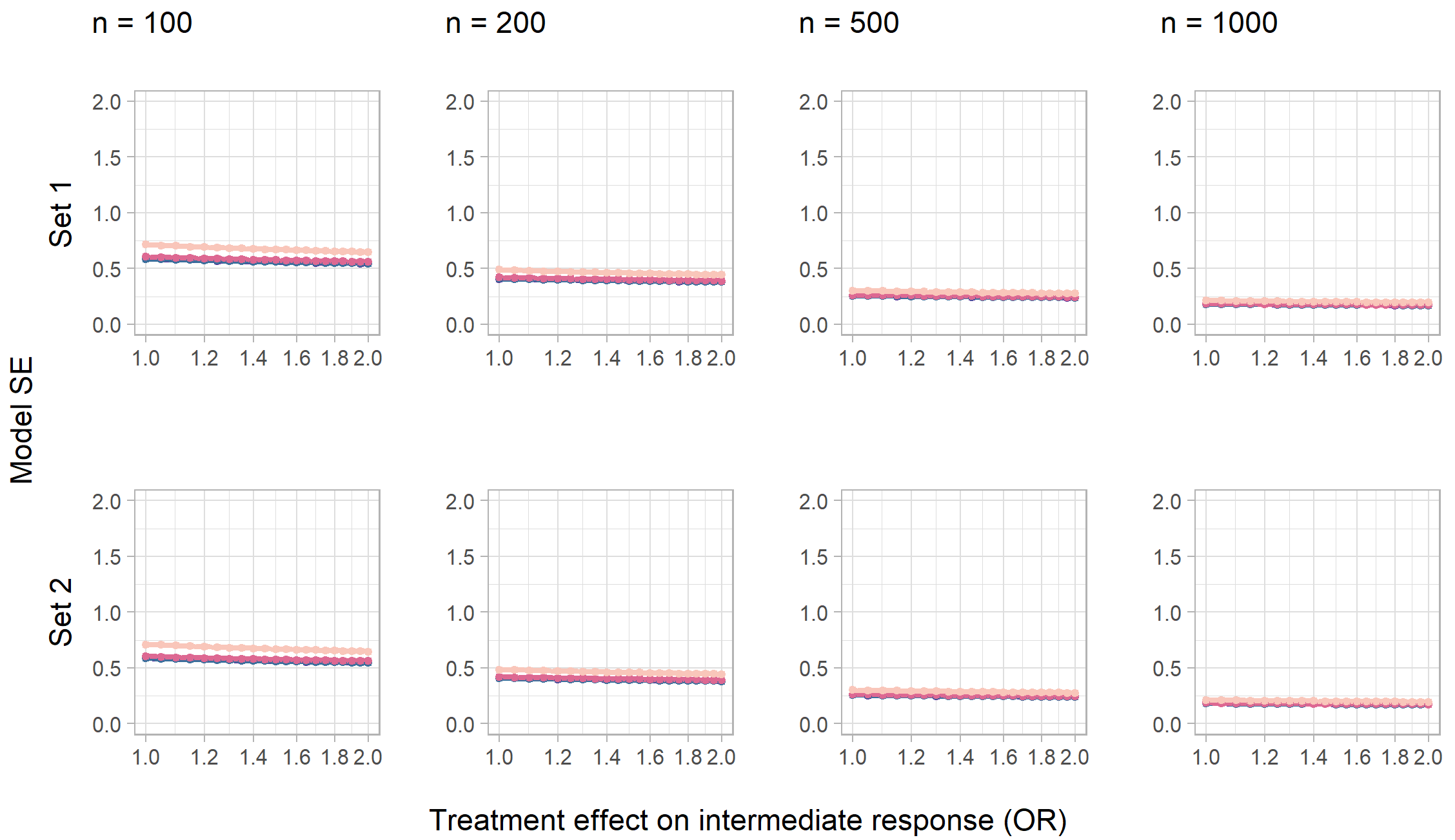


S Figure 39: Model SE in the binary outcome study (sensitivity analysis C) increased event rate). Colour indicates treatment effect on the outcome (ORs) (Purple = 1, blue = 1.2, darkpink = 2, light pink = 5).

## Type 1 error


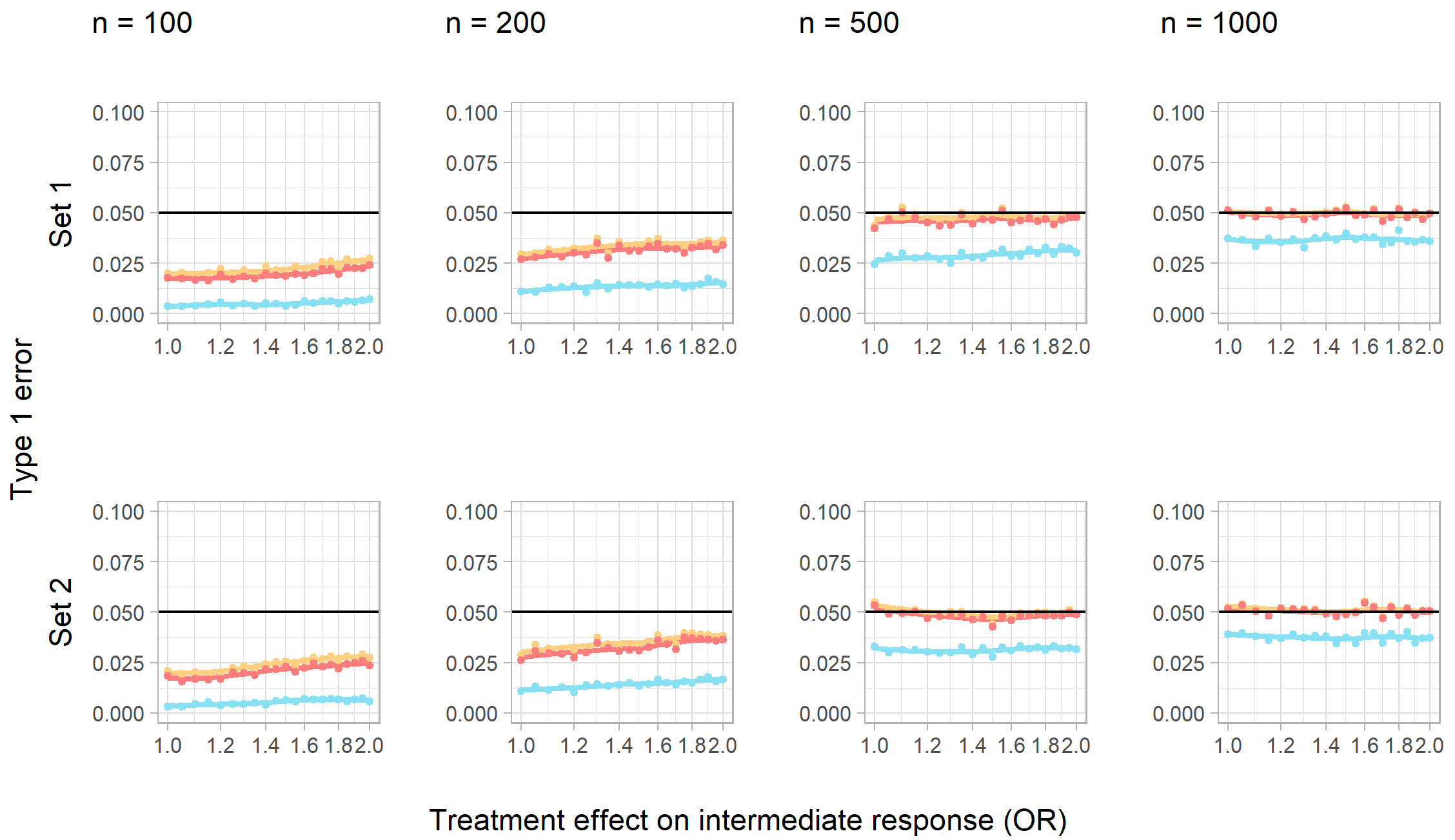


S Figure 40: Type 1 error of statistical tests in the binary outcome study (sensitivity analysis A) increased confounding). Fisher’s exact test = blue, chi-squared test = pink, adjusted chi-squared test = yellow.


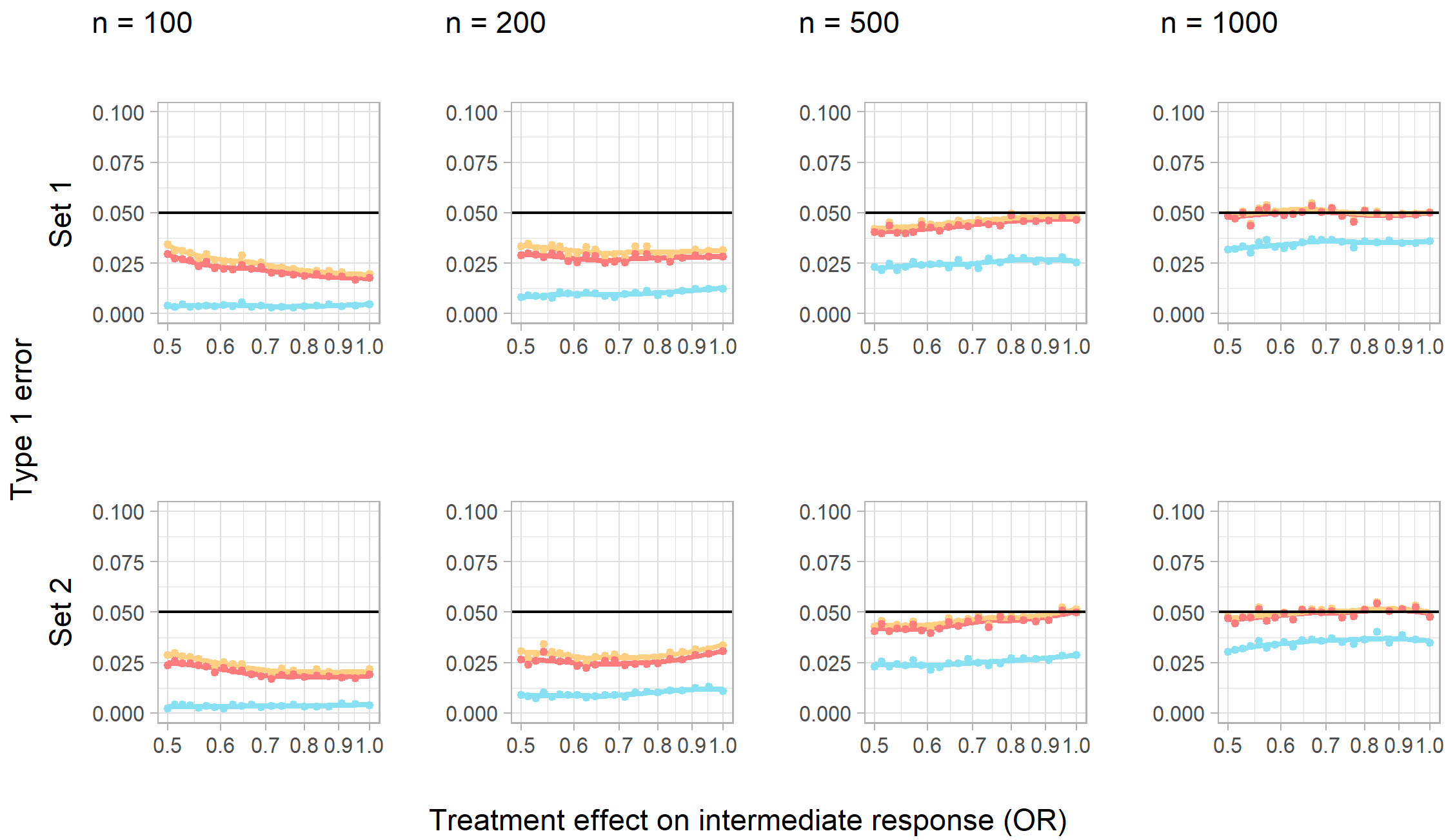


S Figure 41: Type 1 error of statistical tests in the binary outcome study (sensitivity analysis B) changed direction of treatment effect on intermediate). Fisher’s exact test = blue, chi-squared test = pink, adjusted chi-squared test = yellow.


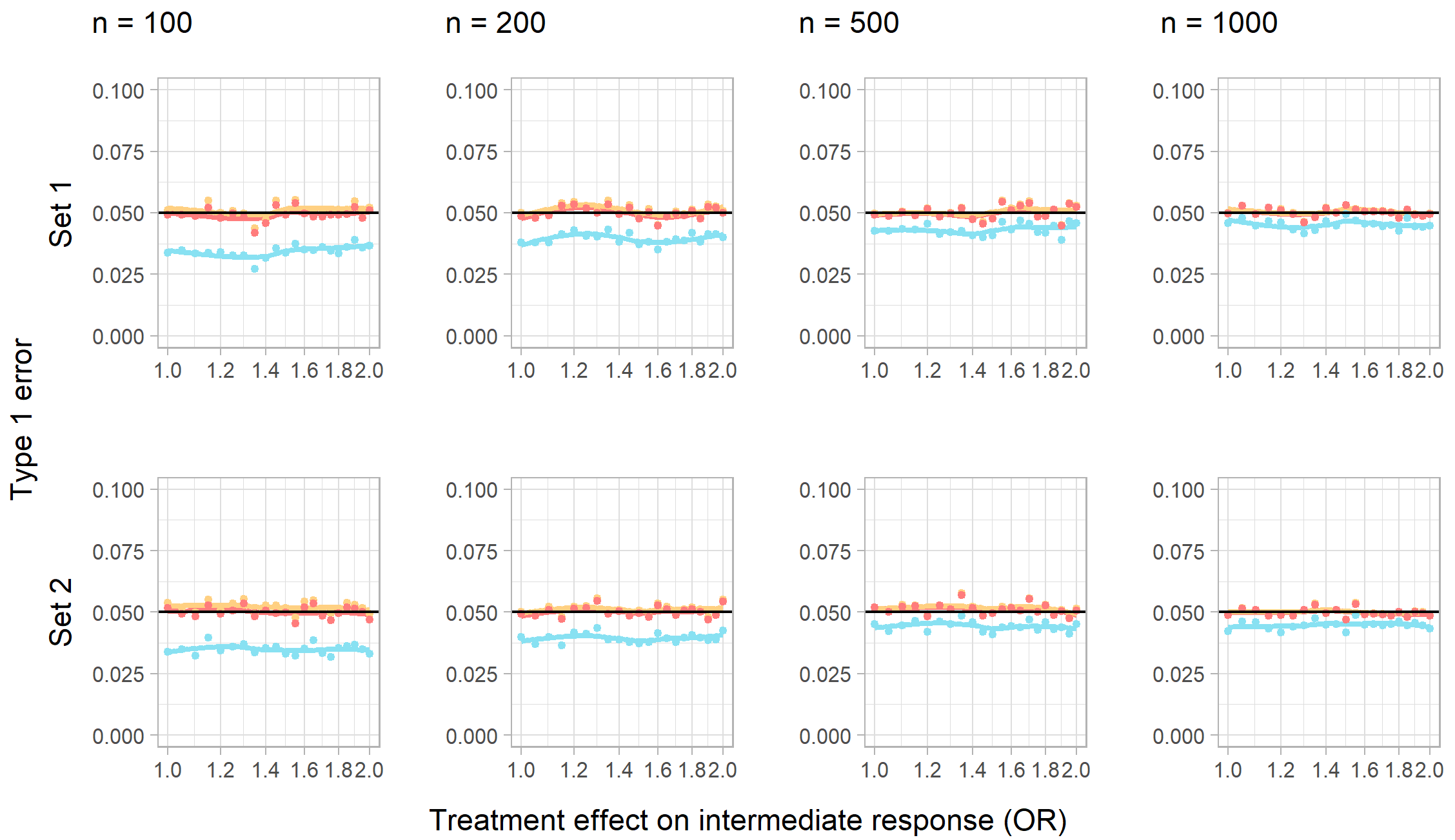


S Figure 42: Type 1 error of statistical tests in the binary outcome study (sensitivity analysis C) increased event rate). Fisher’s exact test = blue, chi-squared test = pink, adjusted chi-squared test = yellow
